# Landscape modification by Last Interglacial Neanderthals

**DOI:** 10.1126/sciadv.abj5567

**Published:** 2021-12-15

**Authors:** Wil Roebroeks, Katharine MacDonald, Fulco Scherjon, Corrie Bakels, Lutz Kindler, Anastasia Nikulina, Eduard Pop, Sabine Gaudzinski-Windheuser

**Affiliations:** 1 Faculty of Archaeology, Leiden University, P.O. Box 9514, 2300 RA Leiden, Netherlands.; 2 MONREPOS Archaeological Research Centre and Museum for Human Behavioural Evolution, Schloss Monrepos, 56567 Neuwied, Germany.; 3 Institute of Ancient Studies, Pre- and Protohistoric Archaeology, Johannes Gutenberg-University Mainz, Schönborner Hof, Schillerstraße 11, 55116 Mainz, Germany.; 4 Naturalis Biodiversity Centre, P.O. Box 9517, 2300 RA Leiden, Netherlands.

## Abstract

Little is known about the antiquity, nature, and scale of Pleistocene hunter-gatherer impact on their ecosystems, despite the importance for studies of conservation and human evolution. Such impact is likely to be limited, mainly because of low population densities, and challenging to detect and interpret in terms of cause-effect dynamics. We present high-resolution paleoenvironmental and archaeological data from the Last Interglacial locality of Neumark-Nord (Germany). Among the factors that shaped vegetation structure and succession in this lake landscape, we identify a distinct ecological footprint of hominin activities, including fire use. We compare these data with evidence from archaeological and baseline sites from the same region. At Neumark-Nord, notably open vegetation coincides with a virtually continuous c. 2000-year-long hominin presence, and the comparative data strongly suggest that hominins were a contributing factor. With an age of c. 125,000 years, Neumark-Nord provides an early example of a hominin role in vegetation transformation.

## Teaser

Studies of ancient lake sites in Germany suggest landscape manipulation by prehistoric hunter-gatherers, 125,000 years ago.

## INTRODUCTION

How far back in time we can observe humans transforming their environments and the spatiotemporal scale and nature of those changes has become a lively research topic, stimulated by debates over the origins and time depth of the Anthropocene [see, e.g., (*1*, *2*)]. Modern humans and their ancestors may have had a substantial impact on ecosystems for a very long time. Some of the ways in which hominins did so are likely to have involved niche construction, the process by which organisms actively modify their own and other species’ niches. Studying this process in the past is challenging: We lack tools for weighing the specific contribution of hominins, herbivores, climate, and other factors (*3*), and it is rarely possible to relate hominin behavior (recorded in time-averaged assemblages) to more detailed and continuous records of environmental change.

Some archaeologists have suggested that forms of ecosystem transformation [sensu (*4*)] go back into prehistory, substantially more than 10 thousand years (ka) ago. Late Pleistocene megafaunal extinctions in which human hunting may have played a role had impacts on vegetation and climate (*5*); local declines in prey populations have been identified earlier (*6*). Other workers have emphasized the impact that early hominins may have had on landscapes by their stone quarrying and stone working activities, from the emergence of stone tool technology onward (*7*). By far, the most examples, however, involve late Pleistocene/early Holocene vegetation transformation through hunter-gatherer use of fire. For example, this has been inferred for the Holocene in the tropical rainforests of Queensland, Australia (*8*), and for the final Pleistocene/early Holocene in western Europe [e.g., (*9*)] as well as for Last Glacial Maximum (c. 23 ka ago) Europe following results obtained via dynamic modeling (*10*).

For island South-East Asia, it has been suggested that pollen, charcoal, and archaeology indicate repeated biomass burning to produce productive forest-edge environments from around c. 50 ka ago onward, shortly after the arrival of the first modern human populations, e.g., at Niah cave, Borneo (*11*). A recent study pushes possible landscape transformation through fire use even further back in time, proposing that early modern humans were deliberately using fire around northern Lake Malawi in Africa to transform a dense, closed-canopy forest into anthropogenic bushy, open-canopy woodland and wooded grassland, clearly visible from around 85 ka ago onward (*12*). The potential global impact of these past landscape modifications is underlined in a recent study, which suggests that human activities have shaped nearly three-quarters of terrestrial nature for at least 12 ka (*4*).

Certain organisms in an ecosystem have an effect on themselves and other organisms that, in proportion to their body size and population density, is relatively large. On the basis of the ethnographic literature, Bliege Bird and Nimmo (*13*) point out that humans can play vital ecological functions comparable to those provided by nonhuman keystone species that exert a major influence on an ecosystem. In “place-based” societies, including hunter-gatherer and other traditional subsistence societies, individuals live and extract and consume resources in the same landscapes and experience feedback from local ecosystems. Over time, interactions between place-based societies and landscapes become fine-tuned through learning and cultural evolution. They identify four main categories of relevant interactions: seed dispersal, fire, bioturbation, and predation. Australian ethnographic studies suggest that fire use has measurable benefits in these contexts (in terms of prey density and habitat diversity) and that these depend on a history of regular burning (*14*). These burning practices by hunter-gatherers are widespread across almost all biomes worldwide and carried out as part of diverse activities (*15*). Further, hunting can influence ecosystem diversity and structure not only through mortality-based suppression of prey populations but also through its contribution to the “landscape of fear” (*16*), to which animals adjust their behavior (*17*). Although not all of these processes are equally archaeologically visible, this is an interesting way in which to think about the environmental impact of past populations, with similar long-term histories of occupation and potential for feedback from ecological interactions.

Here, we address a high-resolution case study of environmental impact by Neanderthals, documented at the Last Interglacial (or Eemian) site complex of Neumark-Nord (Germany), from which we present an integrated record of paleoenvironmental and archaeological data, contextualized within a regional comparison with other contemporaneous lake-basin sites. After a long period of abandonment, when major parts of the northern European plain were covered by late Saalian [Marine Isotope Stage 6 (MIS 6)] ice sheets, hunter-gatherers moved back into this region at the beginning of the Last Interglacial, the Eemian. Where accessible, deposits from this short warm-temperate period of c. 11 ka provide a uniquely rich paleoenvironmental record. In Europe, evidence for hominin activity turns up rather frequently where Last Interglacial sediments are exposed and when investigated by multidisciplinary teams (*18*–*20*). At some of those sites, evidence suggests that hominins were present and intensely active through multiple seasons (*21*). The Eemian is therefore an interesting period in which to explore the way hominin activities and other factors interacted to shape the landscape—even more so, because, given its comparability to the Holocene, it is sometimes used as a possible baseline for conservation studies (*22*).

Identifying the impact of hunter-gatherer activities on a landscape, which entails assessing the role of dynamic climatic, biotic, and anthropogenic factors, is very challenging; as yet, the evidence for the suggested anthropogenic effects in deep prehistory mentioned above is ambiguous at best (*15*). High-resolution paleoenvironmental and archaeological archives with well-established chronologies can be considered as a prerequisite to track a “hominin ecological footprint” in past environments through time. This is particularly important because the proxies used need to be clearly linked to human activities, such as in the case of charcoal particles, which can result from both natural and anthropogenic fires. The challenges also include the generally small number of paleoenvironmental proxies available, discrepancies in spatial scale between environmental reconstructions and hominin activity, and equifinality of hominin activity and other disturbance factors. Here lies the relevance of our case study: The Neumark-Nord site complex provides unique paleoecological and archaeological data documenting a long phase of distinct vegetation openness that correlates with a c. 2-ka-long well-documented history of significant hominin presence in the area. In addition, the Neumark-Nord site complex is located in a region in which several other localities with contemporaneous sediments, exposed through large-scale open-cast lignite mining, have been sampled for relevant proxies. This region is the Mitteldeutsches Trockengebiet, a relatively arid area with low precipitation, situated in the rain shadow of the Harz mountains in central Germany (*23*), which provides good control for comparability of regional climatic conditions, as there are no conceivable differences in rainfall or temperature in this small region. This allows us to focus on the role of other factors, including herbivore presence, basin form, and hominin activity.

Parts of the Neumark-Nord record have already been published in some detail (*21*, *24*–*28*), summarized here in a brief introduction to the geological setting of the site complex and the traces of a Neanderthal presence, focused on those aspects that are relevant for identifying hominin transformation of the local environment around the lakes (see the Supplementary Materials for more details). This is followed by Results in which we first present data on the open and diverse character of the vegetation of the Neumark-Nord lake landscape, which we then proceed to compare to the environmental records from other Last Interglacial lake basin sites in our study area, to weigh the factors contributing to the openness, including climate, basin morphology, herbivores, and hominins. In Discussion, we interpret these results in terms of a specific Neanderthal impact on the environment at Neumark-Nord and beyond.

### The Neumark-Nord sites and neighboring basins

#### The Neumark-Nord sites

**Fig. 1.**
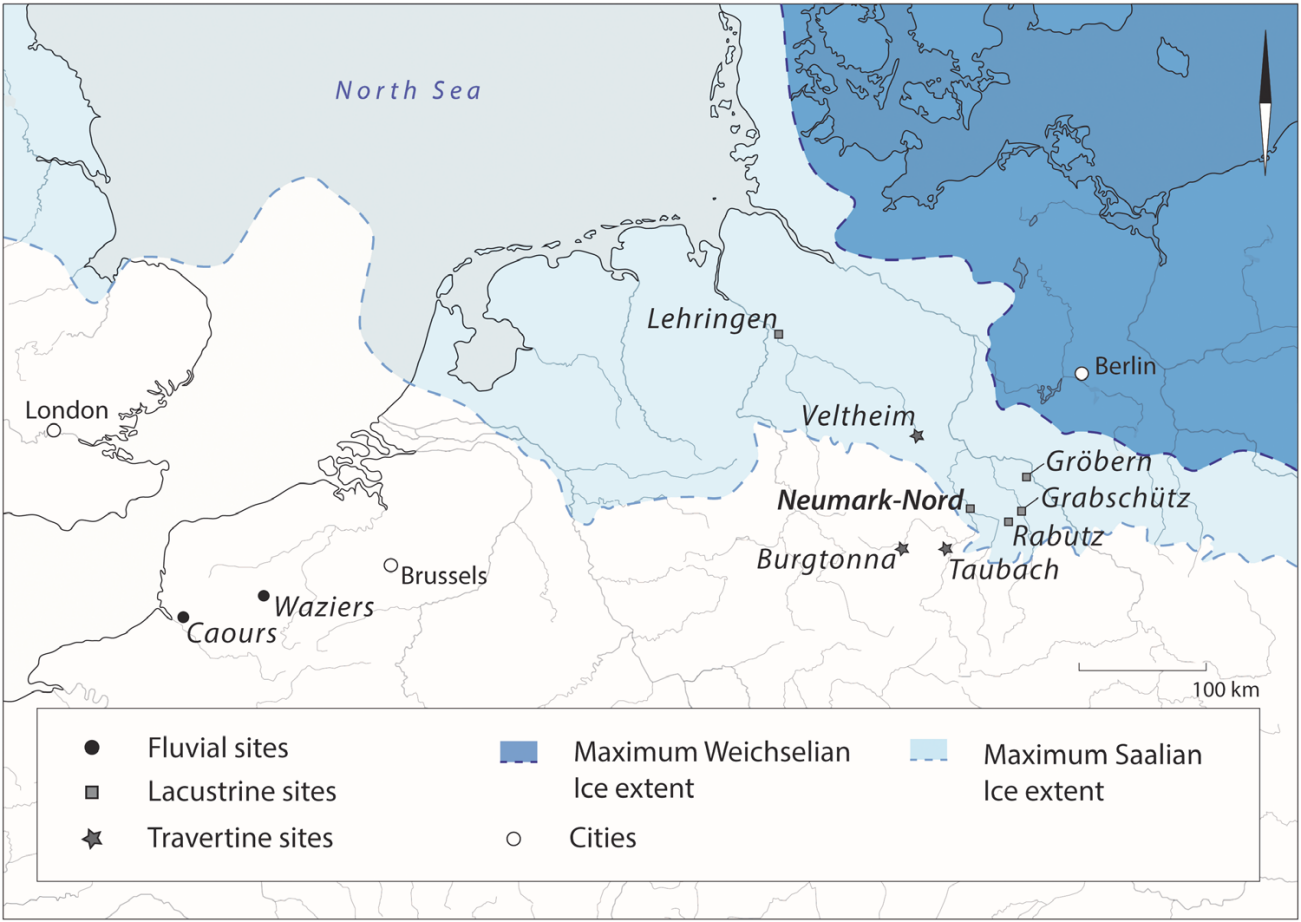
Location of Neumark-Nord and other Last Interglacial archaeological sites relative to the maximum ice extents of the Saalian and Weichselian glaciers. Modified after (*18*).

Neumark-Nord is located about 10 km south of the German city of Halle, in an area that was covered by Saalian ice sheets at the end of the Middle Pleistocene but was not reached by the ice advance of the subsequent Weichselian glaciation (Fig. 1). The location in between the maximum advances of these MIS 6 and MIS 2 glaciers is relevant for understanding the exceptional quality and quantity of well-preserved Last Interglacial sequences in this part of Europe. The retreat of the Saalian glaciers exposed a landscape covered in sediment-receiving structures such as kettle holes, meltwater channels, and other sediment traps caused by a range of glacial and postglacial processes. The water bodies that developed in these landscapes filled up with sediment during the Last Interglacial. Covered by thick wind-blown deposits during the next glacial and not reached by the Weichselian glaciers, these basin infills, where accessible, provide a uniquely rich paleoenvironmental (and sometimes archaeological) record for the Last Interglacial, which is not well represented elsewhere in the European sedimentary record south of the Saalian-glaciated area. The Last Interglacial vegetation succession can be studied here in high chronological resolution, through the identification of individual Last Interglacial pollen assemblage zones (PAZs) (*29*, *30*). The durations of these zones are well known (table S1), calculated on the basis of on varve counting of annually laminated lacustrine sediments at sites in northern Germany, c. 200 km northwest of Neumark-Nord, resulting in a total duration of c. 11 ka for the Eemian in northern Europe (*29*).

The Neumark-Nord Last Interglacial sediments were exposed in the open-cast lignite mine Mücheln, in the Geiseltal valley near Merseburg, Saxony-Anhalt, Germany (51°19′28″N, 11°53′56″E). After the 1985 discovery of the Neumark-Nord 1 basin as a very promising source of paleoenvironmental and archaeological data, the Mücheln exposures were investigated by D. Mania and his team in a continuous rescue operation until the end of mining activities in the mid-1990s (*31*, *32*). The team recorded c. 30 km of sections through the Neumark-Nord deposits (*32*), salvaged countless faunal remains, and documented a vast number of bone and lithic scatters in rescue interventions [see, for example, (*24*, *31*) (fig. S2)]. Their work provided detailed insights into the geology, paleoecology, and archaeology of the Neumark-Nord 1 paleobasin, which was 24 ha at its maximum extension. The basin has become well known for the discoveries of numerous virtually complete skeletons of large mammals, e.g., straight-tusked elephants, rhinos, fallow deer, and aurochs, and an abundance of faunal remains, in general, interspersed with a wide range of archaeological traces of Neanderthal activities around its shores (fig. S2) (*24*, *31*), including close-range hunting of fallow deer (*33*). The basins’ faunal remains are usually very well preserved, which enabled sequencing the full mitochondrial genome and partial nuclear genome of the Last Interglacial straight-tusked elephant *Palaeoloxodon antiquus* (*34*).

The smaller (c. 1.6 ha) Neumark-Nord 2 basin was found by Mania’s team during reclamation works in the abandoned mine complex in the late 1990s. Situated about 100 m northeast of Neumark-Nord 1 (figs. S2 and S3), the Neumark-Nord 2 basin was targeted by a series of multidisciplinary investigations and long-term archaeological excavations between 2004 and 2008 [see (*25*) for a review]. In the text that follows, “Neumark-Nord” refers to the whole of the sampled lake landscape, while “Neumark-Nord 1” and “Neumark-Nord 2” refer to the individual basins and their margins.

Both basin structures formed in Saalian till deposits and subsequently filled up with fine-grained Last Interglacial sediments (*35*): The Neumark-Nord 1 basin’s infill consisted predominantly of silt loams and peat deposits reaching a total thickness of 15 m, while the more clastic infill of the shallow pool Neumark-Nord 2 reached a total thickness of 10 m. A section in the deeper part of the Neumark-Nord 2 basin, Hauptprofil 7 (HP7), was continuously sampled for a multidisciplinary study of the basin infill, which included micromorphology (*36*), pollen analysis (*37*, *38*), paleomagnetism (*28*), small mammal studies (*39*), and malacology (*40*), with a recent study using oxygen and carbon stable isotopes of *Bithynia tentaculata* opercula from the section as a new type of environmental proxy (*41*). The sediments containing the environmental data can be clearly related to the occupations recorded by the archaeological data. At both basins, the interglacial sequence ends with Early Weichselian loess deposits.

The sedimentary processes creating the Neumark-Nord 2 infill and the relationship between the environmental data and the occupations reflected in the archaeological record have been published in great detail elsewhere [e.g., in various contributions in Gaudzinski-Windheuser and Roebroeks (*25*) and by Pop *et al.* (*42*); see also the Supplementary Materials]. The distance between the main profile HP7 and the archaeological excavation area is small (c. 20 m, as visualized in fig. S3), and all layers could be tracked toward the excavation area very accurately. All data indicate a geologically rapid infilling of the small basin, with micromorphological studies (*36*) demonstrating that sedimentation was a nearly continuous process. Calcareous silt loams dominate the infill. These were deposited by overland flow in a very calm sedimentary setting in placid water, with only very short (<1 decade) interruptions during which the depression fell dry. Because of the high sedimentation rate (table S1), traces of bioturbation and incipient soil formation are virtually absent throughout the sequence (*28*, *36*). Energy levels were low, and there is no evidence for transport (>1 m) of artifacts, as even the smallest flint artifacts cluster together with large and heavy lithic manuports (*42*).

On the basis of its stratigraphic position, pollen records, as well as geochronological analyses, which include thermoluminescence dating of heated flint artifacts from find level NN2/2B with a weighted mean age of 121 ± 5 ka, optically stimulated luminescence of sediments, paleomagnetism, and amino acid racemization studies of *Bithynia* opercula, the infill can confidently be attributed to the Last Interglacial (*25*). The Neumark-Nord 1 and 2 pollen records (*38*, *43*, *44*) and the known duration of individual PAZs of the Last Interglacial (table S1) (*28*, *29*) provide great temporal control for finds made in the basin deposits, as well as the means to correlate the deposits of both basins on the scale of specific vegetation zones (*21*, *33*).

The wide range of proxies retrieved from the infill of the two Neumark-Nord basins has contributed to a high-resolution paleoenvironmental record that enables fine-grained positioning of human presence in the larger lake landscape against its floral and faunal background. These correlations also indicate that we are not dealing with independent sedimentary basins but with two water bodies that were part of a much larger Last Interglacial lake landscape that developed after the retreat of the late Saalian (MIS 6) glaciers from this area. A very small part of this landscape was sampled in the Neumark-Nord 1 and 2 exposures and studied in detail within the Neumark-Nord 2 excavated area. During our fieldwork, traces of four more potential basin structures, still buried under loess, were found within 1.3 km of Neumark-Nord 2, an illustration of the lake-dotted character of the MIS 6 glaciated Saale-Elbe area (*45*).

At Neumark-Nord, Neanderthals were distinctly present during Last Interglacial PAZ III and PAZ IV, with a total duration of c. 2 ka (*29*), while only a few traces of their presence have been recorded for the *Carpinus* (hornbeam) phase (PAZ V) of the Last Interglacial (*21*, *33*), with a duration of c. 4 ka (table S1). The sediments associated with the *Carpinus* phase in which closed forests dominated received a comparable amount of archaeological and geological attention in both basins, e.g., reflected in the rich fallow deer assemblage from Neumark-Nord 1, in which our team identified traces of hominin interference [e.g., (*33*)]. However, the signal of hominin presence during this second half of the Eemian is very weak.

**Fig. 2.**
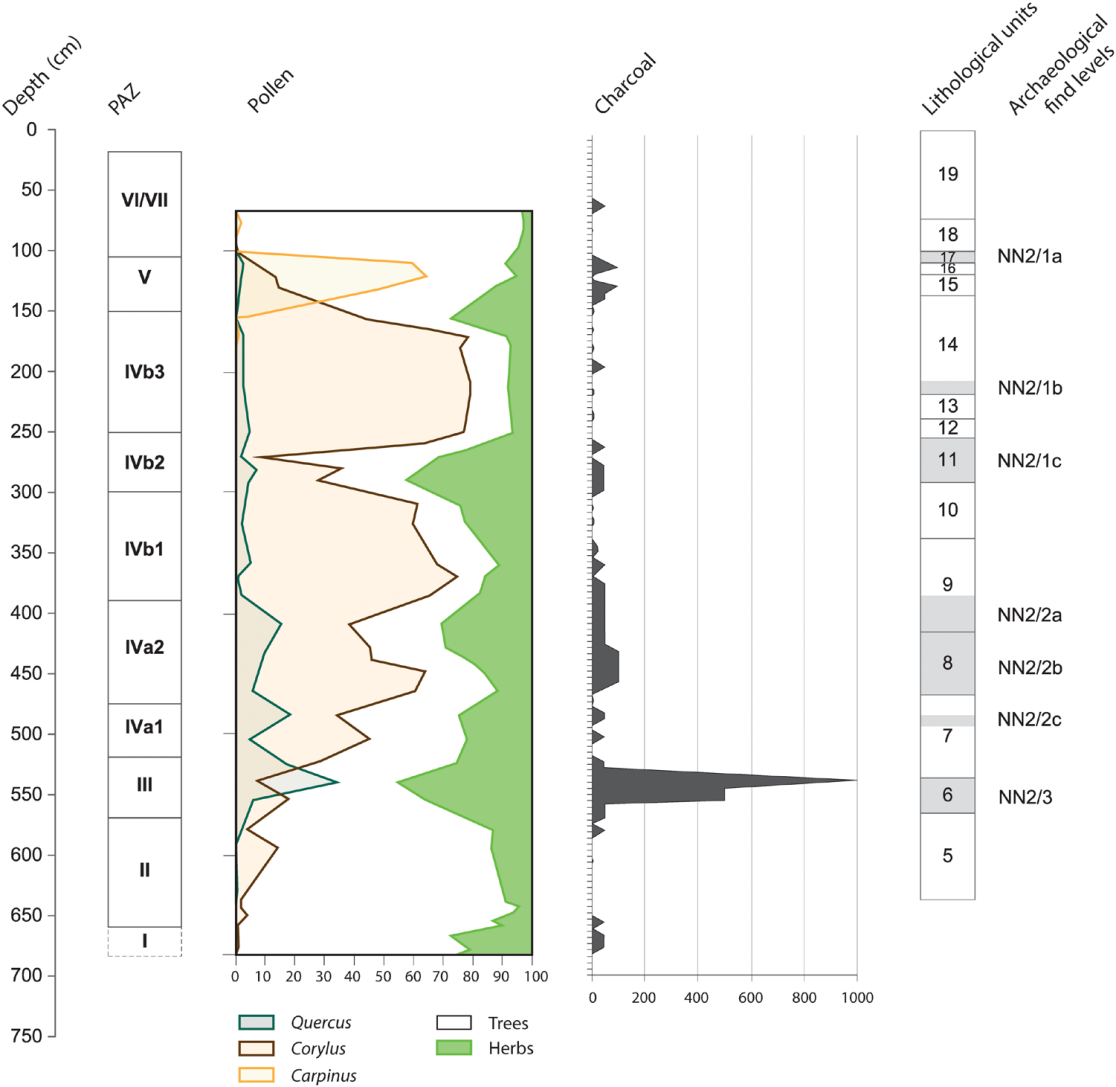
Overview of archaeological and environmental data for Neumark-Nord 2. Neumark-Nord 2, HP 7/10 section, with depth in centimeters, PAZ (for duration in years, see table S1), composite pollen percentage diagram (see also Fig. 3), charcoal particles >1 mm/5 liters of sediment, lithological units, and archaeological find levels. For a detailed description of this section, see Supplementary Text and fig. S1.

Hominin presence at Neumark-Nord is attested by large amounts of stone artifacts and modified bone fragments recovered from the shore areas of the basins, mostly dating to the early (PAZs III and IV) parts of the Last Interglacial (Fig. 2). The excavations of find level 2/2 at Neumark-Nord 2 yielded about 20,000 flint artifacts and more than 118,000 well-preserved faunal remains (*21*) from an excavated area of 491 m^2^, and recovered in primary archaeological context. The heavily cut-marked and fragmented faunal remains represent a minimum number of 166 large mammals, mostly horses [minimum number of individuals 56 (MNI 56)], bovids (MNI 40), and cervids (MNI 53) (*21*). Seasonality data, obtained by analysis of the remains of more than 50 faunal remains from fetal to subadult individuals from four different taxa, demonstrate that Neanderthals were active here all year round (*21*). Together with a virtual absence of carnivore marks on the rich faunal remains from Neumark-Nord 2, this strongly suggests a form of Neanderthal “dominance” or even “permanence” in the lake area during formation of the NN2/2B assemblage (*21*).

The high-density Neumark-Nord 2 find scatter can be contrasted with a “background” signal of low-density, single-activity death or kill sites recorded at Neumark-Nord 1 (*21*, *24*), characterized by remains of large animal carcasses and by large, simple flakes found on the immediate shore of the lake. However, with increasing distance from the lake itself (i.e., 50 to 100 m from the inferred shore), Mania’s team also documented NN2/2B-like, much denser, archaeological scatters around the Neumark-Nord 1 basin in their rescue operations (fig. S2). Formed during PAZ IV (*21*), these scatters are characterized by the increased presence of highly fragmented animal bones, flint knapping waste, flint cores, and charcoal particles, with large chunks of charcoal [Plate 10 in (*24*)] particularly characteristic for these “lake margin facies” distributions (*24*, *31*). Against this background, the Neumark-Nord 2 high-density concentrations can be read as an integral part of a larger Neumark-Nord lake landscape that was extensively exploited by hominins, with the Neumark-Nord 1 and 2 archaeology representing a small sample of the evidence of hominin activities once scattered around this landscape.

The Neumark-Nord 2 excavations yielded substantial evidence for fire use at and around the excavated area, reviewed by Pop *et al.* (*27*). Heated lithics (artifacts and cobbles), heated bone fragments, burned wood, charred seeds, and charcoal particles were uncovered during excavation, while systematic sampling of HP7 in the deeper part of the Neumark-Nord 2 basin (fig. S3) yielded thousands of charcoal particles and more charred seeds (Fig. 2) (*40*).

Most of the charcoal particles retrieved from this section [see (*27*, *40*)] lie within the 1- to 2-mm size range and do not show signs of edge rounding, suggesting that they were not subjected to any substantial movement. The vertical distribution of the macroscopic charcoal of HP7 is shown in Fig. 2. After initial low charcoal concentrations (<10 particles/5 liters) in the birch-pine levels predating the earliest archaeological finds in the basin, Neumark-Nord 2 lithostratigraphic Unit 6 shows a strong peak, the largest one of the HP7 sequence, with densities of 1000 particles/5 liters. All charcoal particles (>1 mm) recovered from the excavated Neumark-Nord 2 area and from the HP7 section submitted to taxonomic analysis originate from deciduous trees, with identified pieces (51.9%) dominated by *Quercus* sp. and cf. *Quercus* sp. (42.4%) (*27*).

Macroscopic particles of charcoal (>200 μm) are usually of local origin, particularly the larger-sized (>500 μm) category, recovered from Neumark-Nord 2 through the use of sieve mesh sizes of 0.5 mm (*27*). They probably derive from fires within the watershed of the basin, although a more distant source, up to several kilometers, is possible on the basis of actualistic experiments [e.g., (*46*)]. It is likely that some of the fires burned close to where the thermally altered lithics and bones were found, 15 to 25 m away from the HP7 sampling location, although, given the ubiquity of hominin activities in the wider area, the charcoal source areas may have been diverse. An anthropogenic origin for the macroscopic charcoal from the basin area (HP7) is supported by the correlations between the artifact and charcoal datasets, described in detail by Pop *et al.* (*27*). The distinctive charcoal peak in Unit 6 correlates with the first documented presence of archaeological finds in the two Neumark-Nord basins, reflecting the arrival of Neanderthals in the wider lake landscape (Fig. 2) during PAZ III of the Last Interglacial. This spike, with 10 times the amount of charcoal particles of any other peak in the Neumark-Nord 2 sequence, coincides with a drop of the upland deciduous forest in general, as well as with a strong rise in the upland herbs curve in the Neumark-Nord 2 sequence. In addition, the 400- to 450-cm part of the sequence, which includes the lower part of the rich archaeological find level of NN2/2B, documents higher levels of charcoal sustained over a longer period. Charcoal was also abundant in the archaeological find levels from the shore area of Neumark-Nord 1 but was not systematically retrieved there (*31*). The recurring presence of fire proxies in the form of thermally altered archaeological material and charcoal particles in the various find levels at Neumark-Nord 2 and 1 indicates that repeated, temporally distinct, fire events took place on or near the gently sloping margins of the basins, during the earlier part of the Last Interglacial.

Interpreting the charcoal record in terms of hominin activities is complex, because we do not know exactly what the source areas were within the Neumark-Nord 2 basin area, and these may well have varied over time. What we can see is that the marked charcoal peak in Unit 6 coincides with the arrival of the first hominins in the wider area, at Neumark-Nord 1 and 2, as reflected by the archaeological record of both basins, including a small assemblage in level NN2/3. The massive hominin occupation—with many on-site proxies for fire use (*27*)—reflected in the rich NN2/2 find levels did not translate into a similar strong peak, illustrating the complexity of the charcoal signal. The notable Unit 6 peak suggests an increase in frequency or scale of fire activity within the Neumark-Nord 2 basin environment. This may indicate one or more natural fires burning heavy fuels within the watershed, a high frequency of or large-scale anthropogenic fires, or a combination of both natural and anthropogenic fires. Roebroeks and Bakels (*47*) already speculated that this peak may reflect anthropogenic burning of parts of the landscape, as it co-occurs with a strong increase in nonarboreal pollen, i.e., an opening up of the landscape, providing space for oak and hazel and their edible hazelnuts and acorns [see also the discussion in (*15*)]. That suggestion is systematically addressed and evaluated in this study, against the background of contemporaneous vegetation successions from the same area.

#### The neighboring basin sites and travertine sites

Among other Last Interglacial lakes in the area, two nearby basins, the sites of Gröbern and Grabschütz (*45*, *48*), respectively, 55 and 32 km northeast of Neumark-Nord, and in their size roughly similar to Neumark-Nord 1, have yielded only very few traces of hominin presence: at Gröbern, a straight-tusked elephant skeleton with a small number (*n* = 25) of associated stone artifacts, and at Grabschütz, a low-density flint scatter, of in total 13 flint flakes. Like the Neumark-Nord sites, these sites were found and studied during systematic surveys of the Pleistocene exposures in the brown-coal quarries of the Saale-Elbe area from the 1970s onward (*45*). Preservation at these sites is broadly comparable to the Neumark-Nord basin sites. Both sites yielded detailed pollen records for the Last Interglacial (*49*, *50*), which enabled comparison with the Neumark-Nord vegetation sequences.

The Neumark-Nord 1 and 2 pollen sequences cover the entire Last Interglacial, and the information provided by these sequences is not restricted to the period of Neanderthal presence there. The Gröbern pollen sequence also covers the whole Eemian, while the Grabschütz diagram only lacks a part of the earliest (PAZ I) Last Interglacial strata (*49*). The arboreal vegetation successions identified at these sites are a perfect fit with the succession established for the Eemian in northern Germany by Menke and Tynni (*30*). This succession is steered by climate, distance to the refugia of the trees, and soil development. Within a restricted area with the same substrate and climate, this succession is the same and contemporaneous. Over large distances, differences are observed, but as Velichko *et al.* [(*51*), p. 218] found in their study of central and eastern Europe: “...the main phases in the evolution of the vegetation appear to have been similar throughout the latitudinal belt under consideration.” This relative homogeneity of vegetation and vegetation succession has been related to the fact that the Eemian was a high sea level interglacial (*52*), notably more oceanic in western and central Europe than the Holocene, indicated, for example, by the frequent occurrence of ivy (*Hedera*) and holly (*Ilex*) in Eemian sequences in western Russia, far to the east of their present distribution.

In the region discussed in our paper, the succession implies the establishment of forests with a closed canopy. Open areas, for instance, caused by forest fires, must have existed, but we can expect these patches to have been evenly distributed over the region and to have returned to forest after a restricted period of time. All vegetation sequences from within the Mitteldeutsches Trockengebiet share the same set of plant species in the same succession with the signal specific to Neumark-Nord consisting of a prolonged and unique period of open vegetation in the first half of the Last Interglacial. It is the absence of conformity to the general pattern and the notable long-term openness of the Neumark-Nord vegetation in combination with a strong hominin presence that triggered our study.

Pollen preservation was rather poor for the rich archaeology-bearing levels at another nearby lake site, the Last Interglacial basin of Rabutz, c. 30 km east of Neumark-Nord and c. 10 km south of Grabschütz, although the presence of other environmental proxies, especially macrobotanical remains, does allow a qualitative comparison with Neumark-Nord (see below). Just south of the Saalian glaciated area with its numerous postglacial lakes, a rich series of travertine sites also document an interglacial presence of hominins, with, e.g., the rich archaeological site of Taubach (Fig. 1) of well-established Last Interglacial age (*53*).

## RESULTS

### Vegetation openness at Neumark-Nord

The basin infill of Neumark-Nord 2 documents the complete vegetation succession of the Last Interglacial, which is, in the usual manner, described by a series of PAZs (*38*). The sequence starts with a dominance of *Betula* (birch) pollen and herbs during PAZ I, followed by a *Pinus* (pine) forest in PAZ II. During PAZ II, the first deciduous trees turn up to replace pine definitively during PAZ III. The resulting deciduous forest is dominated by *Quercus* (oak), but the high percentage of upland herbs shows that this is not a closed-canopy forest. *Quercus* values drop rather early in the sequence. PAZ IV is dominated by *Corylus* (hazel). PAZ V concerns a closed forest dominated by *Carpinus* (hornbeam). In the upper part of the sequence, *Pinus* replaces *Carpinus*.

The documented vegetation succession is typical for the Eemian in western and central Europe (*30*), but the high proportion of herb pollen during PAZ III and PAZ IV is uncommon. The vegetation can be characterized as semiopen in a 1-km radius surrounding the Neumark-Nord basin [catchment area size based on actualistic studies of similar environments by palynologists who compared pollen rain deposition, basin size, and known vegetation, e.g., (*54*)]; the basin margins were covered by grassland, and the pollen shows signals of disturbed/trampled soils, witnessed by, for instance, the presence of *Polygonum aviculare* (knotgrass). This relatively open signature can be seen from PAZ III up to PAZ V. The possibility that the herb pollen grains had their source in animal dung, dropped or washed into the water, has been refuted (*37*).

A comparison with the palynological data from the much larger water body of Neumark-Nord 1 shows that the Neumark-Nord 2 signal of vegetation openness is part of a spatially larger phenomenon: Seifert-Eulen’s (*43*, *55*) pollen curves for Neumark-Nord 1, based on samples located within deeper parts of the basin, show a comparable drop of oak as well as an increase of upland herbs in the same stratigraphic position. Taking into account the taxa represented, the semiopen landscapes would have consisted of patches of both open grassland and bare/disturbed soil vegetation as well as patches of trees: initially of oak and later predominantly hazel [cf. (*38*)].

The landscape stayed open for the whole of PAZ IV, with an estimated duration of c. 2.2 ka (*29*), until the *Carpinus* phase (PAZ V), when the forests closed. As mentioned above, for this c. 4-ka-long closed-forest phase, only an ephemeral hominin presence has been documented at Neumark-Nord, despite dedicated attention to these deposits.

The results of the Neumark-Nord 1 pollen data are supported—be it at a different (smaller) spatial scale—by an abundance of mollusk species characteristic of open environments, accounting for 50 to 70% of all terrestrial specimens and indicating a local dry and warm microclimate, with steppe-like open vegetation around the lakes (*24*, *32*, *56*, *57*). Of the terrestrial species, mollusks characteristic of open environments also dominate the assemblage from the basin infill at Neumark-Nord 2 (*40*).

The composition of the rich mammal fauna from Neumark-Nord (*21*, *24*, *31*), which includes straight-tusked elephant, rhino, wild boar, horse, large bovids, and a range of cervids, as well as lion, hyena, and bear (see table S2), also points to a diverse environment that comprised both forested patches and large open areas (*26*). Although potentially biased by prey preference of Neanderthals (see also below), this interpretation is supported by stable isotope analyses of ungulate faunal remains from Neumark-Nord 2 (*58*).

**Fig. 3.**
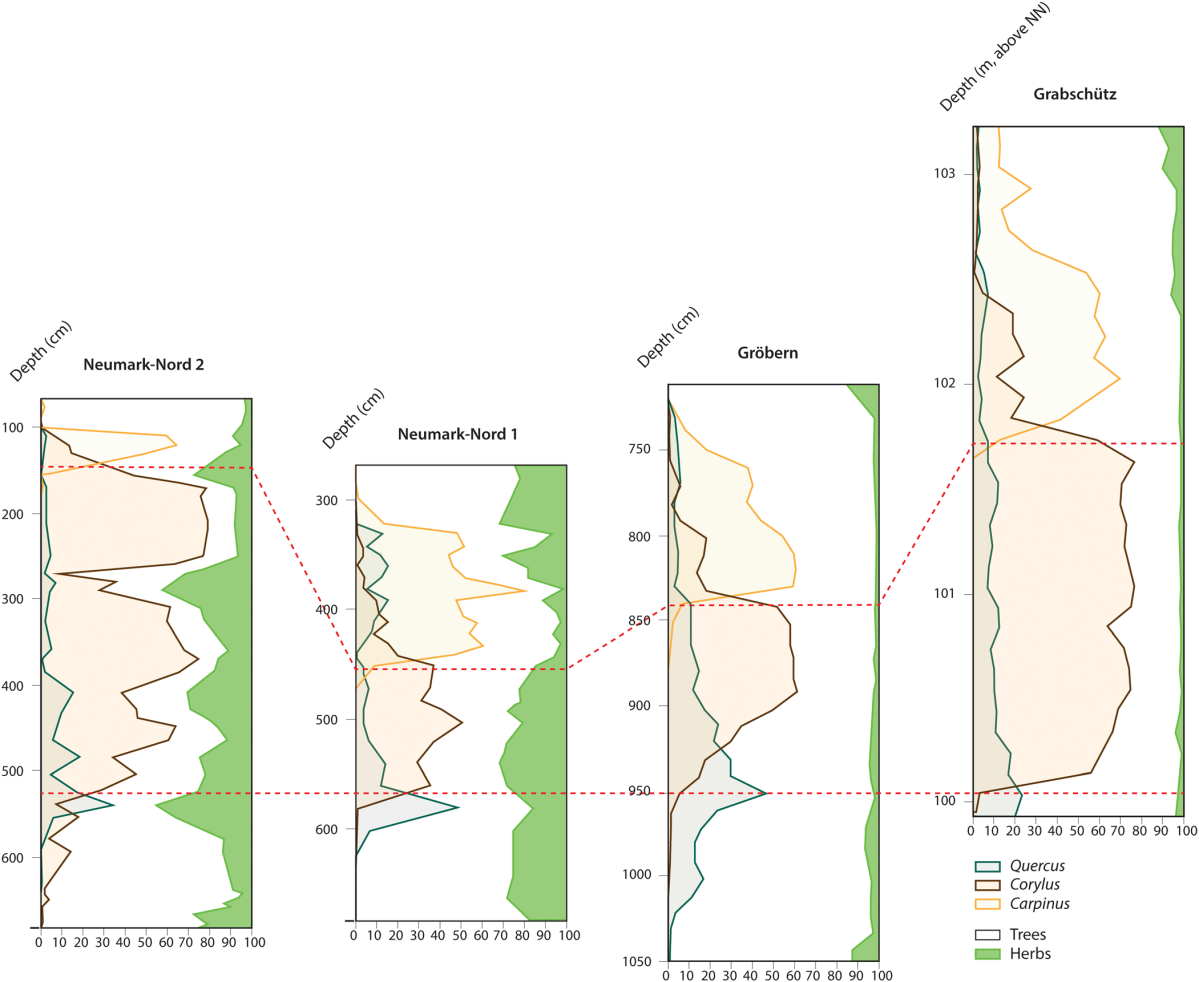
Comparison of Last Interglacial pollen diagrams from Neumark-Nord, Gröbern, and Grabschütz. Composite pollen diagrams with trees and shrubs in white, dryland herbs in green, and the percentage curves for three main defining arboreal taxa, *Quercus*, *Corylus*, and *Carpinus*. The pollen sum is the same for all four diagrams: All plants except aquatics and riverine species. Graphs after (*38*) (recalculated), (*55*), (*62*), and (*50*). Depth in Grabschütz is given in meters above sea level (NN, normal null); the others are floating depths within the sampled sections. The red dashed lines confine PAZ IV as defined in the original diagrams.

Data regarding rainfall and temperature during the period of Neanderthal presence at Neumark-Nord 2 come from stable isotope analysis of freshwater mollusk opercula (*41*) and are in agreement with the results from the oxygen isotope analysis of horse tooth enamel from the Neumark-Nord 2 site (*59*), which has yielded mean annual temperature estimates for the Last Interglacial. The Mitteldeutsches Trockengebiet in which Neumark-Nord is situated has a mean annual temperature of ~9°C (*60*) and is in an area of the German “isoscape” with annual average rainfall δ^18^O values between −8.7 and − 9.4 ‰ [see Fig. 3 of (*61*)]. Data presented by Britton *et al.* (*59*) indicate that the climate at Neumark-Nord 2 during the early Eemian was broadly comparable to today, with similar predicted annual average rainfall d18O values and mean annual temperatures (*59*).

### Comparison with other Last Interglacial sites

This distinct openness of the wider Neumark-Nord landscape forms a contrast to the palynology-based vegetation reconstructions from the two nearby Last Interglacial basins of Gröbern and Grabschütz, which indicate predominantly closed forested conditions [Fig. 3, (*45*, *48*–*50*)]. Our expectation that rainfall and temperature will not differ between sites in the study region is partially confirmed by data from one of the baseline sites, Gröbern, where the temperature history of the Eemian has been reconstructed by palynology and by the bulk carbonate d18O record from that basin (*62*, *63*), both methods indicating very similar temperature histories for Gröbern and Neumark-Nord, with temperatures reaching a maximum at both sites during PAZ IVb and remaining stable during PAZ V [see also (*41*)].

Open space around water bodies can be caused by several factors, a common one being fluctuations in water level. A drop in water level can cause the margins of pools to fall temporarily dry, and such stretches of bare land are quickly invaded by herbs. This results in open spaces lacking trees. The size of the open terrain depends on the steepness of the slope of the basin: The steeper that slope, the narrower the margin, which is apt to fall dry. Furthermore, steep slopes may potentially inhibit large herbivore access. Therefore, the slope of the four basins should be taken into consideration. Data on the morphology of the Neumark-Nord basins (*31*, *35*) and the Gröbern and Grabschütz evidence (*48*) indicate that steeper slopes do not account for the absence of open space around Gröbern and Grabschütz; while slope shape varied notably within the individual basins, the Neumark-Nord 2 slopes ranged between 10^o^ in the south and 30° to 40° in the north, the Neumark-Nord 1 ones between 15° and 40° (*31*, *35*), while for Gröbern slopes of 15° and 30° or even lower are given, and for Grabschütz 7° and 11° (*48*). This implies that factors other than basin morphology explain the distinct open space present at Neumark-Nord.

Large herbivores are also well known to play an important role in vegetation openness through their grazing and browsing and by disturbance through their trampling and earth moving activities [e.g., (*22*)]. While large herbivores were certainly present at all of these sites, the specific mammalian faunal records compared here are variable in terms of numbers of faunal remains, from a handful of large mammal individuals at Gröbern and Grabschütz (*64*) to many hundreds recovered from the Neumark-Nord exposures (table S2) (*21*). They also differ in the genesis of the assemblages, with in the case of Neumark-Nord a very strong anthropogenic input through hunting activities (*21*). Translating these assemblages into former environments calls for dealing with these filters, which is problematic (*64*, *65*). What we can say is that the Last Interglacial large mammal faunas, in general, suggest a forest mosaic, as indicated by wild boar (*Sus scrofa*), cervids (*Cervus elaphus*, *Capreolus capreolus*, and *Dama dama*), and the bovid *Bos primigenius*, and open, steppe-like environments, indicated by horse (*Equus* sp.) and bison (*Bison priscus*). While the (admittedly very small) fauna from Gröbern, which includes straight-tusked elephant, seems to lack any species clearly preferring an “open” environment, horse and bovid are present in the small Grabschütz assemblage, which, like the Gröbern one, might mainly represent a natural death assemblage; horses and bovids dominate in the Neumark-Nord 2 anthropogenic assemblage (table S2).

Considering Neumark-Nord in isolation, herbivore activity has been suggested as the most likely factor to explain the open vegetation, largely based on the herbivore community structure and the sheer quantity of individuals recovered there (*26*). However, death assemblages result from a variety of processes including, in the case of Neumark-Nord, anthropogenic carcass transport rather than simply reflecting the foraging ranges of herbivore populations or their densities; lower quantities of herbivore remains at other basins in the Mitteldeutsches Trockengebiet do not document former herbivore density there. In addition, when addressing the impacts of large herbivores on Eemian vegetation, one must also consider the role of the large carnivores (table S2) in an ecosystem: Predators influence herbivore behavior and density, and by releasing plants from herbivory, they also indirectly affect plant communities [e.g., (*66*, *67*)]. Nevertheless, what matters is that all these basins were within the foraging range of the same large mammal community, which included species preferring closed forest conditions and others preferring more open environments.

The palynological data–based interpretation of unusual vegetation openness at Neumark-Nord during the Last Interglacial finds support in a comparative study of the Last Interglacial land snail faunas from Neumark-Nord with the rich fauna from Burgtonna, a location for which no reliable pollen record exists (*68*). Burgtonna is a travertine site situated 50 km west-southwest of Neumark-Nord, where over the past century a rich collection of floral and faunal remains was assembled (*69*), alongside sparse lithic evidence for human presence. Neumark-Nord and Burgtonna are both situated in the core of the Mitteldeutsches Trockengebiet but display notable differences in their mollusk fauna: In Burgtonna, the land snail species indicate a completely forested landscape during the whole of the Last Interglacial, with hardly any trace of the grasses and herbs of open vegetation and without any of the steppe species (*70*) so dominant at Neumark-Nord. Because the land snail data from Burgtonna were not collected in a comparable, systematic way to that from the Neumark-Nord basins, comparison of diagrams is not possible. Mollusks furthermore inform us about a substantially smaller area than large mammals do, and despite the “closed” character of the local environment indicated by the land snails from Burgtonna, the vertebrate faunal assemblage included most of the large mammal species documented at Neumark-Nord. In terms of distinctly local elements (i.e., excluding mammalian fauna with large foraging ranges), the evidence from Burgtonna, Gröbern, and Grabschütz paints a clear, contrasting picture: one of a relatively closed forest vegetation rather than the relatively open surroundings of the Neumark-Nord basins.

While strongly differing from these three Last Interglacial sites, Neumark-Nord might not constitute a unique case in its specific combination of a rich archaeological record and an environmental signature of distinct and continued vegetation openness: In that respect, there are strong similarities between the Neumark-Nord archaeological record and the one from the Last Interglacial site Rabutz, 30 km east of Neumark-Nord. The Rabutz basin was yielding flint artifacts as early as 1907, together with abundant macrobotanical remains, charcoal particles including charred hazelnuts (*71*, *72*), and heavily fragmented and cut-marked remains of a range of mammals (table S2), including large cervids, rhinoceros, horse, and bison (*65*). The c. 35 large mammal individuals identified in the archaeology-bearing levels formed only a part of the fauna recovered from the site (*65*). Early 20th-century studies of the archaeology-bearing deposits, which included archaeological excavations in 1914 and 1920 and of the associated floral (*71*) and faunal (*65*) remains demonstrated that hominins were repeatedly active there in a pre-*Carpinus*, oak-dominated phase of the Last Interglacial in various seasons and probably over a period of several hundreds of years.

Pollen preservation is poor in the archaeology-bearing deposits (*73*) of the lower part of the Rabutz sequence, and hence, direct palynological comparison to Neumark-Nord, Gröbern, and Grabschütz was not possible. However, the abundant macrobotanical remains in the find layer, studied by Weber (*71*), point to the presence of an open grass landscape, interspersed with “forest islands.” Weber (*71*) was puzzled by the openness of this environment, as in his view, climatic conditions would have allowed closed forests; he even went so far as to suggest that humans ignited fires to keep the landscape open, which in his view would explain the abundance of charcoal fragments, large and small, in the archaeology-yielding 1-m-thick silt deposits. As at Neumark-Nord, forests rapidly took over the Rabutz landscape after the deposition of the archaeology-bearing sediments, as visible in both the macrobotanical remains (*71*) and the pollen data from the *Carpinus* phase of the sequence and upward (*73*).

## DISCUSSION

Despite the uniquely high resolution of the Neumark-Nord 2 record, interpreting the vegetation changes in terms of the specific roles of, for example, herbivores, climate, natural fires, or hominin activities is challenging. The opening up of the Neumark-Nord vegetation as reflected by the rise in upland herbs in PAZ III, the distinct charcoal peak in the lower part of HP7, and the arrival of hominins in the Neumark-Nord area are “simultaneous” events within the sedimentary sequence of Neumark-Nord. However, what does this “simultaneousness” mean? Any correlation between the occupation events that produced archaeological level NN2/3 and the first charcoal peak at Neumark-Nord 2 is constrained within 450 years (the duration of PAZ III) and can be further refined to c. 45 years when considering the sedimentation rates of the encasing matrix, the 5-cm sampling units that yielded the pollen and charcoal samples (table S1). No matter how fine-grained in geological time, this is not fine enough to establish whether Neanderthals moved into the area because it had opened up through natural fires (as, for example, reflected in the charcoal record) or whether the initial suppression of woody vegetation was, in fact, caused by Neanderthal burning activities. It is the time-averaged dimension inherent to archaeological data that causes such equifinality issues, even in high-resolution sites like Neumark-Nord. It is only by ignoring the time dimension of these data that “…is it possible to invoke interpretive theories that are based on ethnographic scale observations of the interactions between individuals and their environments” [(*74*), p.89].

Despite these limitations, the longer time span considered in our study and its larger spatial scale can add to understanding of the factors shaping vegetation openness at Neumark-Nord. In particular, it is our study’s contextualization of the Neumark-Nord data within the evidence from Gröbern, Grabschütz, Burgtonna, and Rabutz that makes for the compelling hypothesis that Neanderthals were responsible for maintaining open habitats at Neumark-Nord. Our starting point is that the continued openness of the vegetation coincides with a virtually continuous c. 2-ka-long distinct hominin presence in the small Neumark-Nord 2 window and in the wider Neumark-Nord area, an openness contrary to what would be expected on the basis of the general vegetation pattern in the region. The comparative data from Gröbern and Grabschütz strongly suggests that the open character of the Neumark-Nord vegetation cannot be explained solely in terms of climatic factors, edaphic characteristics, basin morphology, and/or herbivore communities and their activities (*26*): All these sites share similar climatic conditions, soil conditions, and basin form, and all were within the foraging range of a large mammal community, which included species preferring closed forest conditions and others preferring more open environments.

At the Gröbern and Grabschütz sites, the pollen data indicate a closed, forested environment, while mollusks suggest a comparable situation for the travertine area of Burgtonna. At these sites, the environment remained closed throughout the Last Interglacial sequence, despite the presence of browsing and grazing animals around these water bodies. The findings at these closed sites agree with Svenning’s (*22*) review of Last Interglacial environments, which pictures “…closed forest as the general upland and lakeside vegetation, although with localized occurrence of open, herb-dominated vegetation.” Similarly, Litt (*75*) states that nowhere in central Europe did the presence of herbivores lead to a considerable opening of forest vegetation, as shown by the composition and the proportion of herb pollen. In his view [(*75*), p. 55], reconstructions of park-like half-open forest meadows during the Eemian lack any scientific support. It is very unlikely that herbivores alone would have initiated and maintained open vegetation at Neumark-Nord for the documented c. 2-ka-long period.

The correlation between a long-term and intensive presence of hominins in the Neumark-Nord area and a c. 2-ka period of distinct vegetation openness is, to some degree, also observable at Rabutz, although the archaeological and paleoenvironmental data there are of lower quality than the Neumark-Nord record. Nevertheless, there too, vegetation openness characterized a long period of hominin presence, visible through stone artifacts, fragmented and cut-marked faunal remains, and charcoal, with forests only closing in the *Carpinus* phase. At this time, many of the postglacial lakes in the area may already have been silted up: At Neumark-Nord 2 for instance, the *Carpinu*s phase (PAZ V), with an estimated duration of 4 ka, is represented by only 50 cm of sediments, whereas the preceding c. 3 ka of the Last Interglacial is reflected in 6 m of sediments (fig. S1 and table S1) (*28*).

Given their prolonged and distinct presence at Neumark-Nord, it is probable that Neanderthals created and maintained a certain vegetation openness, simply by their semipersistent presence around these water bodies, by trampling and clearing vegetation during activities in the shore areas. These activities included hunting and game processing (*21*), lighting fires, collecting flint and other rocks from the Saalian tills for their lithic technology, and gathering wood for fuel and for making tools like spears and digging sticks, and possibly for building structures. Repetitive lighting of campfires around the lakes as well as other small-scale burning activities and the hunting of game animals may, over time, have reshaped vegetation structure and ecological communities in the area, in ways that, over multiple generations, increased the food resources available.

Whether the burning activities reflected in the charcoal particles, charred seeds, and heated lithics and bones also resulted from deliberate use of fire at the landscape scale, i.e., whether they were intended to open up parts of the landscape, is impossible to establish. Any burning of the landscape, whether accidental or intentional, would have increased not only herbivore prey presence but also the plant food yield of the area, as postfire vegetation is usually characterized by various herbaceous taxa, which may include edible ones, and may grow well in response to increased availability of plant nutrients.

While the butchering of large herbivores created a highly visible archaeological record in the Neumark-Nord landscape and at Neanderthal sites in general, nutritional studies strongly suggest that these hominins could not have survived on terrestrial game only: Plants must have played an important role in Neanderthal diets, providing carbohydrates and some of the required nutrients and calories (*76*). A range of recent studies suggest that starch-rich foods were already important before the split between the Neanderthal and modern human lineages and document consumption of a similarly wide range of plant species by Neanderthals across their geographical range (*76*, *77*). If, as the archaeozoological data suggest, Neanderthals were indeed present during most parts of the year in the Neumark-Nord area, supplementing their animal-based diets with plant foods would have been an important part of their subsistence activities. The few charred remains of hazelnut, acorn, and blackthorn (sloe plum) retrieved during excavations at Neumark-Nord 2 (*40*) may reflect such plant food. The increase in upland herbs and grasses, with representatives of the Triticeae tribe, including the wild relatives of wheat and barley, must have enabled easy access to grass seeds, now well established as a widespread component of the Neanderthal diet (*76*). Our main point here is that, whether or not Neanderthals played a role in opening up the vegetation, these conditions would have been to their advantage in terms of a wide range of useful and necessary resources, potentially attracting them to the area and possibly encouraging them to contribute to maintaining these conditions.

The multidisciplinary studies of the Neumark-Nord exposures have highlighted the archaeological visibility of a persistent Neanderthal presence in this lake landscape associated with and, we hypothesize, playing a distinct role in environmental changes around 125 ka ago. This evidence has no parallels in the archaeological record of Neanderthals and is older than parallels in the record of *Homo sapiens*. This is not unexpected: To identify hominin modification of prehistoric landscapes, we need long and high-resolution geological sequences with abundant paleoenvironmental and archaeological proxies. These sequences are extremely rare, particularly when we look further back in time; furthermore, one also needs comparative data from contemporary sequences, obtained here from studies of other exposures in our lake-dotted study area, and/or longer sequences in which relationships between climate and vegetation can be established.

Recently reported multiproxy data from Lake Malawi in southern-central Africa (*12*) also fulfill these conditions and suggest an important role of fire in the transformation toward open vegetation conditions there from 85 ka ago onward, in a period in which archaeological evidence for hominin activity also increased. Because of a direct overlap between archaeological, paleoenvironmental, and charcoal records, and lack of correlation with climate, early modern humans are considered as the producers of (most of) these fires, which are inferred to have kept the landscape open for tens of thousands of years. The Neumark-Nord case study is on a much smaller scale than the Lake Malawi one but substantially richer and more fine-grained in terms of paleoenvironmental data, chronological resolution, archaeological materials, and evidence for the anthropogenic character of the fires that produced the charcoal. Both cases provide a tantalizing glimpse of a role for hunter-gatherer activities in creating more open vegetation at a relatively early date. At Neumark-Nord, it was Neanderthals, not *H. sapiens*, who authored the transformation of the vegetation.

The Neumark-Nord data might also be of relevance for our views on other aspects of the Neanderthal niche. The inferred low population densities and the highly mobile lifestyles of Neanderthals might lead one to expect low archaeological visibility both at the local scale and at larger scales, e.g., of northern Europe. However, traces of their presence are often recovered where Last Interglacial sediments are exposed and studied by multidisciplinary teams that include archaeologists, as is well documented for the wider area around Neumark-Nord discussed here (*21*), and increasingly also in northwestern France (*19*). This could suggest locally higher hominin densities and a less mobile lifestyle during interglacials than thus far assumed, certainly around high-productivity “magnet” locations. While we detected no natural features or unique qualities that explain why Neumark-Nord acted as a focus or magnet for Neanderthal activity, it seems possible that the presence of cultural materials and changes to the landscape attracted people repeatedly to this locality, effectively making it a Paleolithic “persistent place” (*78*). The Last Interglacial travertine site of Taubach, c. 54 km southeast of Neumark-Nord, near Weimar, constitutes another magnet location in the region; Bratlund’s study of the faunal assemblage from the site, only a fraction of what was originally recovered, demonstrated repeated hunting of (mainly young) rhinoceroses (MNI 76), bears (MNI 52), and other large mammals, a successful hunting strategy that was in use for a long period of time (*53*).

What we were able to demonstrate here is that, in the earlier phase of the Last Interglacial, the Neumark-Nord landscape harbored a c. 2-ka-long substantial history of Neanderthal activity, which coincided with a notably continuous vegetation openness over the same period. We hypothesize that the wide range of Neanderthal uses of that landscape, including use of fire, created a visible impact on the vegetation and possibly also on its faunal communities. To test and further refine this hypothesis requires novel approaches to the analysis of paleoenvironmental proxies in archaeological and “geological” excavations in our study area and beyond. This is especially badly needed for the fire record, e.g., by making regular and standardized recording of charcoal frequencies standard practice in Paleolithic excavations as well as in paleobotanical studies. Ideally, these approaches would be included in (re)investigation of Eemian sediments from the sites discussed here or some of the other, as yet unstudied, basins in our study region. In addition, new types of analyses, such as the use of simulation tools to explore the relationship between natural and anthropogenic drivers of fire regimes, to identify human burning in empirical sedimentary records (*79*) look promising, while ancient DNA from various source materials, including sediments, could be integrated in the simultaneous study of multiple taxa through space and time for a more comprehensive understanding of ecosystem-wide changes (*79*, *80*).

The evidence presented here for Neanderthal landscape modification points to an important and thus far unknown aspect of Neanderthal behavior and is older than somewhat comparable evidence from the record of *H. sapiens*. Our hypothesis that the wide range of Neanderthal uses of the Neumark-Nord landscape, including use of fire, created a visible impact on the vegetation, and our multiproxy, comparative study should stimulate new work to address the nature, scale, and distribution of early human impact on ecosystems in the deep past.

## MATERIALS AND METHODS

### Palynological site comparison

The interpretation of the Neumark-Nord data in terms of a Neanderthal impact on vegetation is based on two principles. The first is that the identification and weighing of factors influencing vegetation requires the availability of a baseline: A set of data on natural vegetation conditions used as a control. The second is that climate and substrate are the main steering agents behind vegetation and determine its ultimate nature on the basis of the extant plant species. Interpretation of the data according to these principles requires the availability of high-resolution paleoenvironmental data from locations that provide information on the vegetation without a Neanderthal presence (baseline) as well as sites with clear traces of Neanderthal occupation. Moreover, these sets of data should come from a region with the same substrate, be restricted to the same climatic conditions, and cover the same biozone.

We identified two suitable baseline localities in the region east of the Harz Mountains (Germany), close to the Neumark-Nord site complex. At Gröbern and Grabschütz, pollen diagrams come from basins with only very sparse traces of Neanderthal activities on the basin shores: These two therefore provide baseline data (*49*, *50*). The two baseline localities and Neumark-Nord 1 and 2 are located within short distances of each other (see Fig. 1) and meet the requirements of a restricted area with one type of substrate and an identical climate.

High-quality pollen diagrams that document the vegetation, and are equivalent in quality to those from Neumark-Nord 1 and 2 (*37*, *38*, *55*), are available for the Gröbern and Grabschütz Last Interglacial basins (*49*, *50*).

Before the pollen diagrams from these sites could be compared, it was necessary to check whether the calculations providing the graphs were based on the same set of pollen types, because these are percentage diagrams. Some pollen types are commonly left out of the total number, which provides the hundred percentiles (known as pollen sum) shown in these diagrams, to avoid distortion by the overrepresentation of locally shed pollen. This concerns riparian plants and aquatics. The pollen sum in the original Neumark-Nord 2 publications turned out to differ from the sum in the other three, by excluding all herb pollen types that might not have been shed by plants growing in a dry environment. This diagram was recalculated to match the other three diagrams and to thus make the pollen records from the four basins entirely comparable (Fig. 3).

Other than pollen, paleoenvironmental data from mollusks and fauna are available from several sites in the region; however, because this was not collected systematically except at Neumark-Nord, direct comparison is not possible. Charcoal was only systematically collected at Neumark-Nord 2.

## Supplementary Materials

### This PDF file includes:

Supplementary Text Figs. S1 to S3 Table S1 and S2 References
