# Supplementary material for: Landscape modification by Last Interglacial Neanderthals
Source: Sci Adv. 2021 Dec 15;7(51):eabj5567. doi: 10.1126/sciadv.abj5567 (PMC8673775; doi:10.1126/sciadv.abj5567)
Supplement: Supplementary file 1 — Supplementary Text Figs. S1 to S3 Table S1 and S2 References [file sciadv.abj5567_sm.pdf]

Science Advances
AAAS

# Supplementary Materials for

## Landscape modification by Last Interglacial Neanderthals

Wil Roebroeks*, Katharine MacDonald, Fulco Scherjon, Corrie Bakels, Lutz Kindler, Anastasia Nikulina, Eduard Pop, Sabine Gaudzinski-Windheuser

*Corresponding author. Email: w.roebroeks@arch.leidenuniv.nl

Published 15 December 2021, *Sci. Adv.* **7**, eabj5567 (2021)
DOI: 10.1126/sciadv.abj5567

**This PDF file includes:**

Supplementary Text
Figs. S1 to S3
Tables S1 and S2
References

## Supplementary Text

Description of Profile HP7/10

A key section in the geological studies of the Neumark-Nord 2 exposures is *Hauptprofil* (HP) 7, one of the many sections described inside and around the excavated site. This section was sampled for a wide range of dating and palaeo-environmental techniques. All samples were collected from the very same part of the section, enabling a direct comparison of the results on a 5 cm stratigraphic sampling interval over the entire sequence, up to 600 cm below the top of the section. Stratigraphically lower samples were taken from neighbouring sections within the excavation area, excavation square section 210/296-297 (for palaeomagnetic samples), and HP10 (for palynological samples), 8 m from HP7, stratigraphically partly overlapping and underlying the sampled base of HP7. The *Pinus-Betula* phase in this HP10 section revealed such a good overlap with the HP7 section that the results of both sections may be presented as one single pollen diagram (see (*28*, *38*)).

At the Neumark-Nord 2 site, the basin infill is underlain by a late Saalian (Drenthian) sandy-gravelly till (unit 1 in Fig. S1) and overlain by ca. 6 metres of last glacial (Weichselian) loess. The basin infill starts with sediments reworked from the till (unit 2 in Fig. S1), overlain by a series of mostly well-drained, moist, calcareous silt loams, which have been divided into several sublayers (units 3-19), described below, from the bottom of the sequence going towards the top of the exposed sediments at HP7, and using FAO guidelines for soil profile description (*81*). At the Neumark-Nord 2 site, data on the sediments underlying the silt loams (i.e. sedimentary units 1-2, total thickness c. 300 cm) were mainly obtained by augering. The numbering scheme adopted below refers to the layer numbers in Figure 2 of the paper and to Fig. S1, the Lithological Units. A micromorphological study of the HP7 sequence, based on undisturbed thin section samples, has been published by Mücher (*36*).

1. Sandy loamy diamicton (till), with gravel and well-rounded quartz and porphyry particles, dark grayish-brown in colour, up to 150 cm thick, with a more yellowish-brown top (at HP7/10 only observed through augering).

2. Series of loamy and sandy deposits with gravel particles, occasionally laminated sand layers, up to 175 cm thick (at HP7/10 only observed through augering) interpreted as reworked from the underlying till.
3. Light-brown to brown (7.5 YR 5/4-6/4) silt loam, calcareous, 50 cm thick, structureless, very friable, no cutans, no cementation, no pores, no stone and mineral fragments, no mineral nodules, nature of boundary with horizon below not observed.
4. Olive black (5Y 3/1) silty clay, moist, calcareous, 0-50 cm thick, structureless, abrupt and smooth boundary with horizon below.
5. Dark gray (5Y 4/1), moist, calcareous structureless silt loam, c. 70 cm thick, friable to firm, few distinct sharp brown (7.5YR 5/8) iron mottles; vertical reddish yellow (7.5 YR 6/6) infillings of cracks with silt loam and bands of silt; very few, very fine, discontinuous, in ped tubular, channels; very few very small shell fragments; abrupt and smooth boundary with horizon below.
6. Pinkish gray to brown (7.5YR 6/2-5/2, moist), calcareous, structureless, silt loam, 35 cm thick, very friable; very few, fine, faint, pink (5YR 7/4) iron mottles; darker rectangular and rounded lumps of soil material with lignite, diameter resp. 4x10 and 2x2 cm; lower part contains bone fragments, flint artefacts and mollusc shells; abrupt boundary with horizon below. One undisturbed thin section sample: M3lo-2.
7. Yellowish brown (10YR 5/4), very fine laminated, sub-horizontal, moist, calcareous silt loam, 65 cm thick, very friable to friable, and pinkish gray (7.5YR 6/2) silt on top (2 cm thick); at the bottom, 10 cm thick, a very dark gray (5YR 3/1) calcareous organic band; very few fine iron mottles, reddish yellow (5YR 6/6) with distinct sharp boundaries; with very prominent laminae; one wedge infilling, 11 cm deep and 2 cm wide; abrupt and smooth boundary with horizon below. With bone fragments and flint artefacts. Three undisturbed thin section samples: M3lo-5 in top organic layer; M3lo-4 in very fine laminated silt loam; M3lo-3 in bottom organic layer; thick, very friable; very few, fine, faint, pink (5YR 7/4) iron mottles; darker rectangular and rounded lumps of soil material with lignite, diameter resp. 4x10 and 2x2 cm; lower part contains bone fragments, flint artefacts and mollusc shells; abrupt boundary with horizon below. One undisturbed thin section sample: M3lo-2.

8. Yellowish brown (10YR 5/4) moist, calcareous, structureless, silt loam, 50 cm thick, very friable, very few reddish yellow (5YR 6/8) medium iron mottles with distinct and sharp boundaries; abrupt and smooth boundary with horizon below – main find level, numerous bone fragments and flint artefacts. One undisturbed thin section sample M3lo-6.
9. Brown (10YR 5/3), calcareous, structureless, silt loam; 80 cm thick, friable to firm; very few, small to large, hard, irregular, flat, black, calcareous nodules; boundary with horizon below abrupt and smooth. Few bone fragments and flint artefacts. One undisturbed thin section sample: M3u-1.
10. Yellowish brown (10YR 5/4), calcareous, medium to coarse, subangular blocky, silt loam, 50 cm thick, firm; very few gravel-sized quartz particles, fresh, angular and rounded; very few bones and lithics; abrupt and smooth boundary with horizon below. One undisturbed thin section sample: M3u-2.
11. Very pale brown (10YR 7/3) calcareous, structureless, silt loam, 30 cm thick, very friable; very few, gravel-sized quartz, fresh, angular and rounded; few artefacts and bones; abrupt and smooth boundary with horizon below. One undisturbed thin section sample: M3u-3.
12. Pale brown (10YR 6/3) calcareous, structureless, silt loam; 20 cm thick; very friable; few, very fresh, rounded and angular, rock fragments; very few artefacts and bones; abrupt and smooth boundary with horizon below. One undisturbed thin section sample: M3u-4.
13. Light gray to light brownish gray (2.5Y 7/2-2.5Y 6/2), calcareous, structureless, silt loam, 20 cm thick, friable; many fine, prominent, sharp, reddish yellow (7.5YR 7/8) iron mottles; very few, very fine, discontinuous, oblique, inped, tubular, simple pores; very few artefacts and bones; very few, very fine, black roots; abrupt and smooth boundary with horizon below. One undisturbed thin section sample: M3u-5 in calcium carbonate band.
14. Light gray (10YR 7/2), calcareous, structureless, silt loam, 80 cm thick, friable; very few, gravel-sized (< 0.5 cm in diameter), rounded fresh quartz particles; common, medium to coarse, prominent, sharp, reddish yellow (7,5YR 7/6) iron mottles; they occur also along vertical cracks; in sub-horizontal bands at 45 and 65 cm from horizon bottom, very few, large (up to 2 cm in diameter), hard, and rounded, white calcium carbonate nodules occur; in the top a gypsum layer; very few artefacts and bones; very few pedotubules with pinkish white (7.5YR 8/4),

loose infilling of silt; abrupt and smooth boundary with horizon below. One undisturbed thin section sample: M3u-6 in upper gypsum band.

15. Dark reddish gray (5YR 4/2), lower part structureless, silty clay loam, 20 cm thick, in upward direction platy silty clay loam, friable to firm; organic material increases towards the top of the horizon, the organic matter is laminated, with modern roots; common coarse prominent, sharp, brown (10YR 4/2) iron mottles; sharp and smooth boundary with horizon below. One undisturbed thin section sample: M3u-7.
16. Pinkish white (7.5YR 8/2) calcareous, structureless, silt loam, 5-10 cm thick, ("Seekreide") firm; it is covered by a 2 cm thick, laminated, dark reddish brown (5YR 3/2) organic material; abundant molluscs and mollusc fragments; few recent roots; abrupt and smooth boundary with horizon below. One undisturbed thin section sample M3u-8.
17. Olive brown (2.5Y 4/4) calcareous, structureless, silt loam, 10 cm thick, friable to firm; few, fine to medium, random, inped, tubular pores (root channels?); very few bone fragments and flint artefacts and few molluscs fragments. Abrupt and smooth boundary with horizon below. One undisturbed thin section sample: M3u-9.
18. Light yellow brown (10YR 6/4) calcareous, structureless, silt loam, 30 cm thick, very friable; many medium, distinct and sharp, strong brown (7.5YR 5/8) iron mottles; few shell fragments; clear and smooth boundary with horizon below. One undisturbed thin section sample: M3u-10.
19. Yellow (10YR 7/8), moist, calcareous, structureless, loamy sand, more than 70 cm thick (top not observed) with locally intercalated sand layers; few medium, distinct and sharp, strong brown (7.5YR 5/8) iron mottles; consistency: loose to soft. Abrupt and smooth boundary with horizon below.

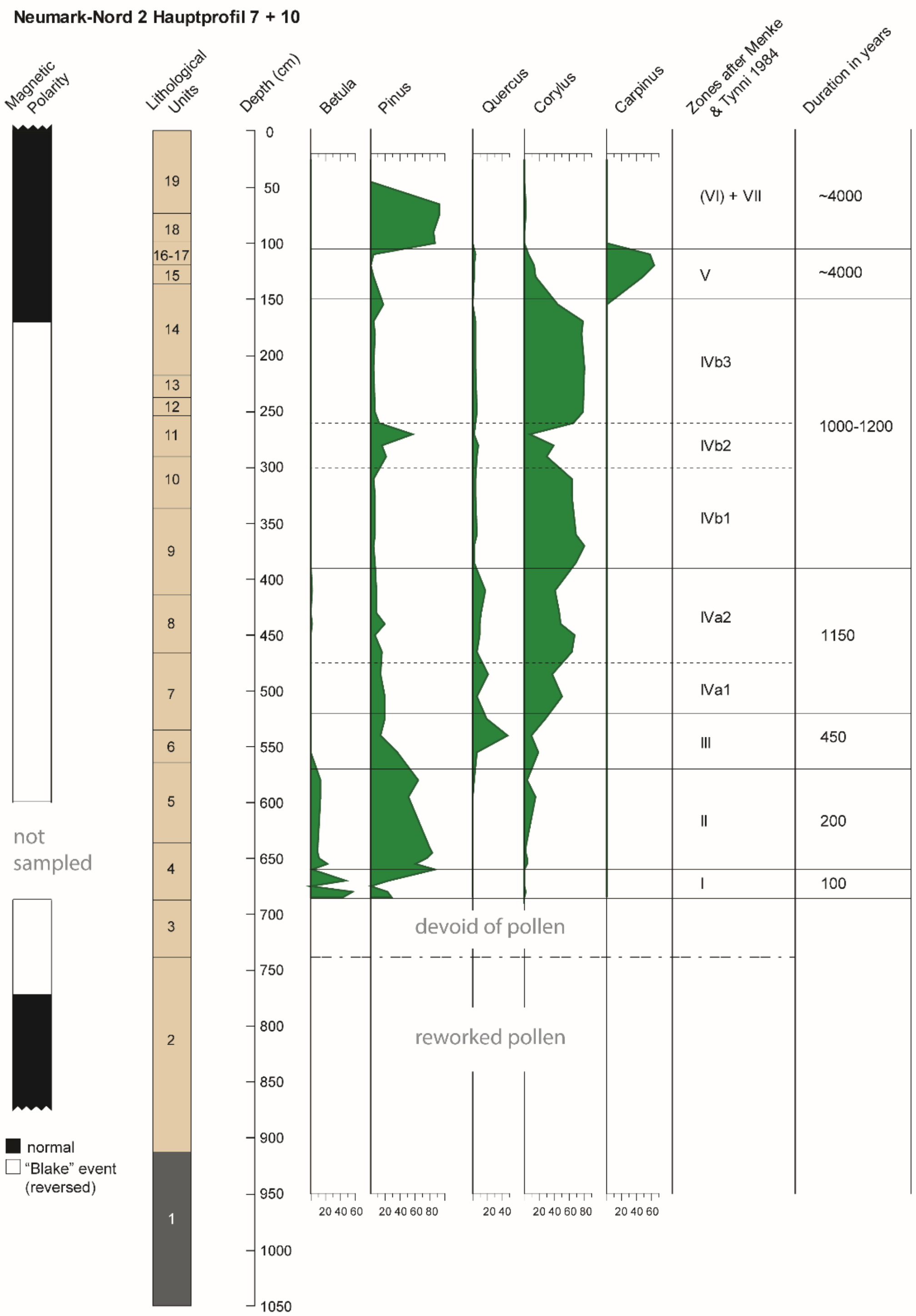
Neumark-Nord 2 Hauptprofil 7 + 10
Magnetic Polarity
Lithological Units
Depth (cm)
Betula
Pinus
Quercus
Corylus
Carpinus
Zones after Menke & Tynni 1984
Duration in years
(VI) + VII
~4000
V
~4000
IVb3
IVb2
1000-1200
IVb1
IVa2
1150
IVa1
III
450
II
200
I
100
devoid of pollen
reworked pollen
not sampled
normal
"Blake" event (reversed)

Figure S1

**Figure S1**

**Combined overview of the stratigraphic and pollen data from HP7/10**. The top of the section is at 101.467 m NN (= above sea level). For description of this profile (including the lithological units 1 to 19) see Supplementary Materials text (Description of Profile HP7/10). The column on the left shows the position of the palaeomagnetic Blake Event in the section (*28*). For data on the duration of the pollen zones and sedimentation rates see Table S1. The pollen percentages shown here are the percentages of the original pollen sum, not of the pollen sum used in the figures in the main text (see Materials and Methods). Figure modified after (*28*).

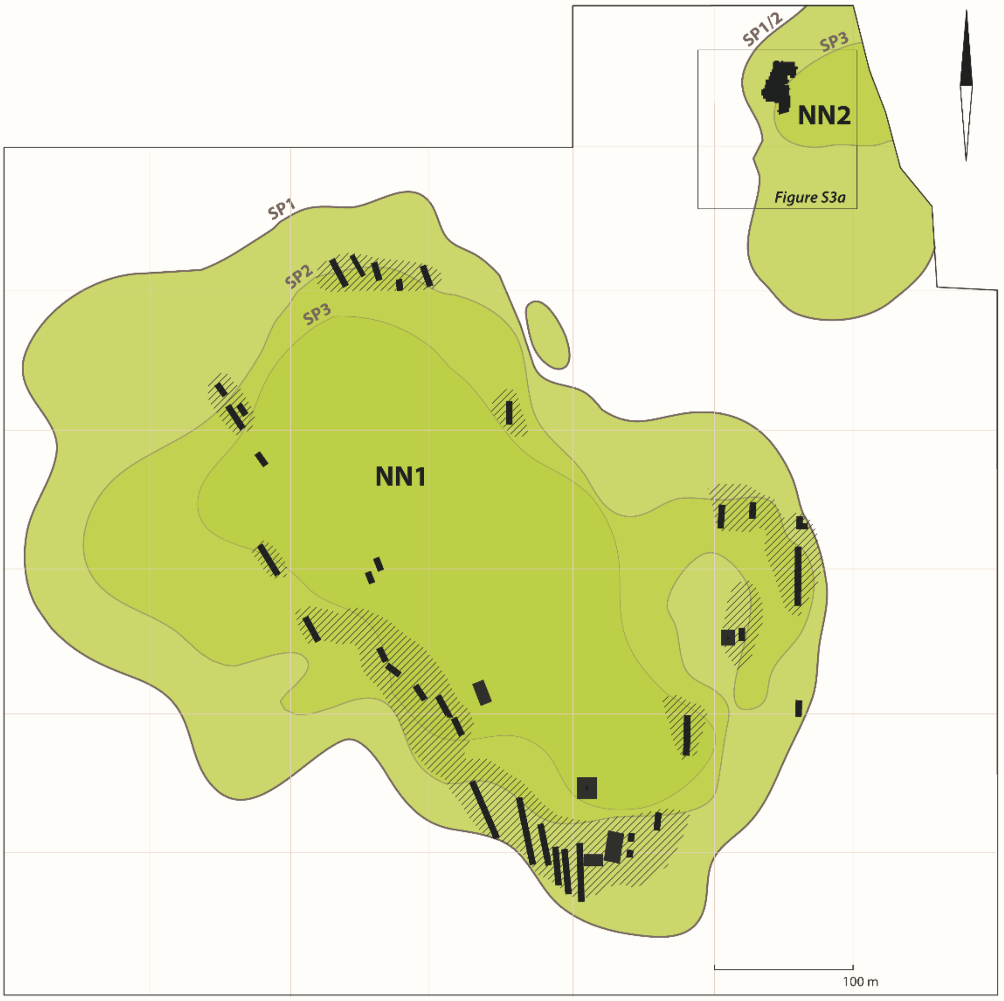


**Figure S2**

**Topographic map of the basin Neumark-Nord 1** in the center of the figure, and the small Neumark-Nord 2 basin in the upper right corner (inset, see Fig. S3 for a close-up), with indicated the locations of the Neumark-Nord 1 archaeological rescue interventions (black rectangles), the high density distributions of flint artefacts and fragmented faunal remains in Neumark-Nord 1 (hatched areas), and the position of the Neumark-Nord 2 excavated area (in black). SP1, 2 and 3: contour lines of the basins, at respectively 8, 16 and 24 m below the surface. SP 1 indicates the maximum extension of the water body. Based on (*21*), with data derived from (*31*), Fig. 38; (*82*).

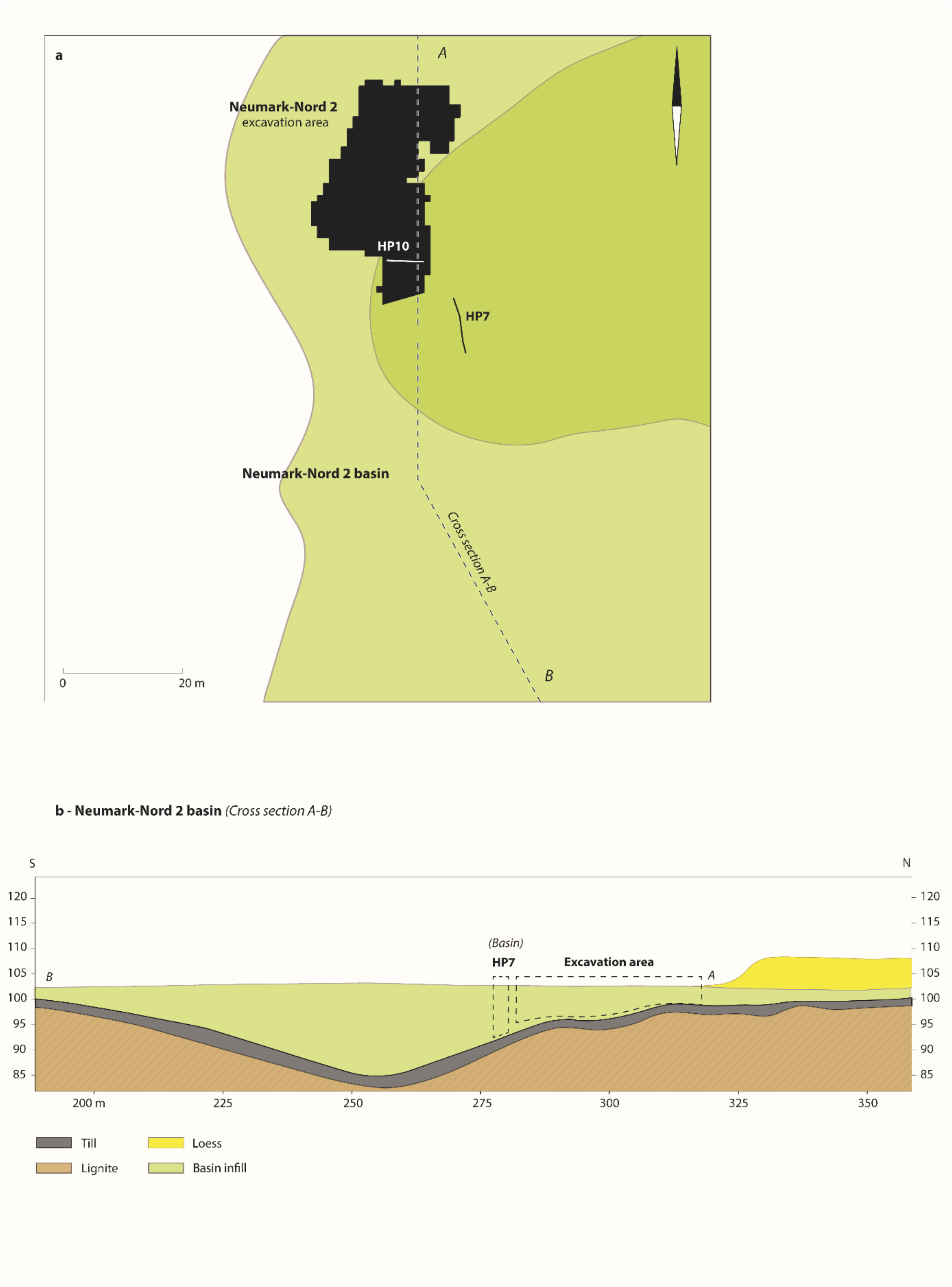
a
A
Neumark-Nord 2
excavation area
HP10
HP7
Neumark-Nord 2 basin
Cross section A-B
B
0
20 m
b - Neumark-Nord 2 basin (Cross section A-B)
S
N
120
115
110
105
100
95
90
85
B
(Basin)
HP7
Excavation area
A
200 m
225
250
275
300
325
350
Till
Loess
Lignite
Basin infill

**Figure S3**

**Figure S3**

**Approximate outline of the Last Interglacial Neumark-Nord 2 basin**, with location of the excavated area and of sections HP7 and HP10. A cross-section of the intersecting (dashed) line A-B is shown below (b), with the basin infill present between the basal Saalian till and the overlying Weichselian loess deposits. The eastern boundary of the Neumark-Nord 2 basin was removed during earlier quarrying activities. Modified after (*42*), Figure 2.

**Table S1**

Sedimentation rate in cm $yr^{-1}$ for NN2 HP 7/10 for individual pollen assemblage zones, based on sediment thickness at NN2 (see Fig. S3) and duration of the Eemian pollen zones as counted/estimated (*) at the Bispingen site (*29*).

| Pollen assemblage zone (*41*) | Duration (in years) (*40*) | Sediment thickness in cm NN2 (see Fig. S1) | Sedimentation rate (cm $yr^{-1}$) NN2 HP7/10 |
|---|---|---|---|
| VI + VII *Pinus* | 4000* | 60 | 0.012 |
| V *Carpinu*s | 4000* | 50 | 0.01 |
| IVb *Corylus* | 1000-1200 | 240 | 0.24 - 0.20 |
| IVa *Quercus-Corylus* | 1150 | 130 | 0.11 |
| III *Pinus-Quercus* | 450 | 50 | 0.11 |
| II *Pinus-Betula* | 200 | 90 | 0.45 |
| I *Betula* | 100 | 30 | 0.33 |

## Table S2

**Neumark-Nord 1 (NN1) and 2 (NN2), Rabutz, Gröbern and Grabschütz.** Identified large mammal taxa and Minimum Numbers of Individuals (MNI), presented per Pollen Assemblage Zone (PAZ) or combinations thereof where information on stratigraphical provenance was insufficient for assignment to specific PAZ. See Table S1 for duration of PAZ. Compiled on the basis of data in: (*21*, *25*, *31*, *45*, *64*, *65*, *72*, *82*).

| **Taxon** | **PAZ I-III** | | | **PAZ IV** | | | | **PAZ I-V** | **PAZ V** | **VI-VII** |
|---|---|---|---|---|---|---|---|---|---|---|
| | NN1 | NN2 | Rabutz | NN1 | NN2 | Rabutz | Gröbern | Grabschütz | NN1 | NN1 |
| **Carnivores** | | | | | | | | | | |
| *Panthera leo* | | | 1 | 3 | 1 | 1 | | | | |
| *Panthera* sp. | | | | | 1 | | | | | |
| *Crocuta crocuta* | | | | 2 | 1 | | | | | |
| *Ursus arctos* | | | | 1 | 8 | 1 | | | | |
| *Canis lupus* | | | | 1 | 1 | 1 | | | | |
| **Megaherbivores** | | | | | | | | | | |
| *Palaeoloxodon antiquus* | 4 | | 1 | 61 | 3 | 1 | 1 | 1 | 7 | |
| *Stephanorhinus kirchbergensis* | | | 6 | 3 | | 5 | | | | |
| *Stephanorhinus hemitoechus* | | | | 3 | | | | | | |
| *Coelodonta antiquitatis* | | | | 3 | | | | | | |
| *Rhinoceros* sp. | | | | 4 | 2 | | 1 | 1 | | |
| **Equids** | | | | | | | | | | |
| *Equus* sp. | | 1 | 1 | >3 | 58 | 1 | | 1 | | |
| **Bovids** | | | | | | | | | | |
| *Bos primigenius* | | | 3 | 10 | | 4 | | | 2 | |
| *Bison* sp. | | | 1 | 1 | | 1 | | | | |
| *Bos/Bison* | | | | | 42 | | | 1 | | 1 |
| **Cervids** | | | | | | | | | | |
| *Capreolus capreolus* | | | | | | 2 | | 2 | | |
| Cervid - *Capreolus* size | | | | | 2 | | | | | |
| Cervid - *Capreolus-Dama* size | | | | | 3 | | | | | |
| *Dama dama* | 1 | | | 26 | | | 1 | | 96 | |
| Cervid - *Dama* size | | | | >3 | 7 | | | | | |
| Cervid - *Dama-Cervus* size | | | | | 10 | | | | 8 | |
| *Cervus elaphus* | 2 | | | 6 | | 9 | 1 | 2 | 24 | |
| Cervid - *Cervus* size | | | | >3 | 28 | | | | | 1 |
| Cervid *Cervus- Alces -Megaloceros* size | | 1 | | | 3 | | | | | |
| *Alces* sp. | | | | | 1 | 1 | | | | |
| *Megaloceros giganteus* | | | 1 | | | 1 | | | | |
| Cervid – *Alces- Megaloceros* size | | | | 2 | 4 | 4-5 | | | | |
| **Pigs** | | | | | | | | | | |
| *Sus scrofa* | | | | | 1 | 1 | | | | |

## REFERENCES AND NOTES

1. N. Boivin, A. Crowther, Mobilizing the past to shape a better Anthropocene. *Nat. Ecol. Evol.* **5**, 273–284 (2021).

2. S. L. Lewis, M. A. Maslin, Defining the Anthropocene. *Nature* **519**, 171–180 (2015).

3. D. R. Braun, J. T. Faith, M. J. Douglass, B. Davies, M. J. Power, V. Aldeias, N. J. Conard, R. Cutts, L. R. G. DeSantis, L. M. Dupont, I. Esteban, A. W. Kandel, N. E. Levin, J. Luyt, J. Parkington, R. Pickering, L. Quick, J. Sealy, D. Stynder, Ecosystem engineering in the Quaternary of the West Coast of South Africa. *Evol. Anthropol.* **30**, 50–62 (2021).

4. E. C. Ellis, N. Gauthier, K. Klein Goldewijk, R. Bliege Bird, N. Boivin, S. Díaz, D. Q. Fuller, J. L. Gill, J. O. Kaplan, N. Kingston, H. Locke, C. N. H. McMichael, D. Ranco, T. C. Rick, M. R. Shaw, L. Stephens, J.-C. Svenning, J. E. M. Watson, People have shaped most of terrestrial nature for at least 12,000 years. *Proc. Natl. Acad. Sci. U.S.A.* **118**, e2023483118 (2021).

5. Y. Malhi, C. E. Doughty, M. Galetti, F. A. Smith, J.-C. Svenning, J. W. Terborgh, Megafauna and ecosystem function from the Pleistocene to the Anthropocene. *Proc. Natl. Acad. Sci. U.S.A.* **113**, 838–846 (2016).

6. J. D. Speth, J. Clark, Hunting and overhunting in the Levantine late Middle Palaeolithic. *Before Farming* **3**, 1–42 (2006).

7. M. Petraglia, in *Human Dispersal and Species Movement: From Prehistory to the Present*, N. Boivin, M. D. Petraglia, R. Crassard, Eds. (Cambridge Univ. Press, 2017), pp. 90–118.

8. P. Roberts, A. Buhrich, V. Caetano-Andrade, R. Cosgrove, A. Fairbairn, S. A. Florin, N. Vanwezer, N. Boivin, B. Hunter, D. Mosquito, G. Turpin, Å. Ferrier, Reimagining the relationship between Gondwanan forests and Aboriginal land management in Australia's "Wet Tropics." *iScience* **24**, 102190 (2021).

9. J. A. A. Bos, R. Urz, Late Glacial and early Holocene environment in the middle Lahn river valley (Hessen, central-west Germany) and the local impact of early Mesolithic people—Pollen and macrofossil evidence. *Veg. Hist. Archaeobot.* **12**, 19–36 (2003).

10. J. O. Kaplan, M. Pfeiffer, J. C. A. Kolen, B. A. S. Davis, Large scale anthropogenic reduction of forest cover in Last Glacial Maximum Europe. *PLOS ONE* **11**, e0166726 (2016).

11. C. O. Hunt, D. D. Gilbertson, G. Rushworth, A 50,000-year record of late Pleistocene tropical vegetation and human impact in lowland Borneo. *Quat. Sci. Rev.* **37**, 61–80 (2012).

12. J. C. Thompson, D. K. Wright, S. J. Ivory, J. H. Choi, S. Nightingale, A. Mackay, F. Schilt, E. Otárola-Castillo, J. Mercader, S. L. Forman, T. Pietsch, A. S. Cohen, J. R. Arrowsmith, M. Welling, J. Davis, B. Schiery, P. Kaliba, O. Malijani, M. W. Blome, C. A. O'Driscoll, S. M. Mentzer, C. Miller, S. Heo, J. Choi, J. Tembo, F. Mapemba, D. Simengwa, E. Gomani-Chindebvu, Early human impacts and ecosystem reorganization in southern-central Africa. *Sci. Adv.* **7**, eabf9776 (2021).

13. R. Bliege Bird, D. Nimmo, Restore the lost ecological functions of people. *Nat. Ecol. Evol.* **2**, 1050–1052 (2018).

14. R. Bliege Bird, D. W. Bird, B. F. Codding, C. H. Parker, J. H. Jones, The "fire stick farming" hypothesis: Australian Aboriginal foraging strategies, biodiversity, and anthropogenic fire mosaics. *Proc. Natl. Acad. Sci. U.S.A.* **105**, 14796–14801 (2008).

15. F. Scherjon, C. Bakels, K. MacDonald, W. Roebroeks, Burning the land: An ethnographic study of off-site fire use by current and historically documented foragers and implications for the interpretation of past fire practices in the landscape. *Curr. Anthropol.* **56**, 299–326 (2015).

16. J. W. Laundré, L. Hernández, P. L. Medina, A. Campanella, J. López-Portillo, A. González-Romero, K. M. Grajales-Tam, A. M. Burke, P. Gronemeyer, D. M. Browning, The landscape of fear: The missing link to understand top-down and bottom-up controls of prey abundance? *Ecology* **95**, 1141–1152 (2014).

17. R. Bliege Bird, C. McGuire, D. W. Bird, M. H. Price, D. Zeanah, D. G. Nimmo, Fire mosaics and habitat choice in nomadic foragers. *Proc. Natl. Acad. Sci. U.S.A.* **117**, 12904–12914 (2020).

18. W. Roebroeks, B. Speleers, in *Le Dernier Interglaciaire et les occupations humaines du Paléolithique moyen*, A. Tuffreau, W. Roebroeks, Eds. (Centre d'Etudes et de Recherches Préhistoriques; Université des Sciences et Technologies de Lille, 2002), pp. 31–40.

19. D. Hérisson, J. Locht, L. Vallin, L. Deschodt, P. Antoine, P. Auguste, N. Limondin-Lozouet, S. Lefebvre, G. Hulin, B. Masson, B. Ghaleb, C. Virmoux, in *Proceedings of the European Society for the study of Human Evolution*, *Vol. 5* (2015); https://hal.archives-ouvertes.fr/hal-02333459, p. 115.

20. S. Wenzel, Neanderthal presence and behaviour in central and Northwestern Europe during MIS 5e. *Dev. Quat. Sci.* **7**, 173–193 (2007).

21. L. Kindler, G. M. Smith, A. García Moreno, S. Gaudzinski-Windheuser, E. Pop, W. Roebroeks, in *Human Behavioural Adaptations to Interglacial Lakeshore Environments,* A. García-Moreno, J. M. Hutson, G. M. Smith, L. Kindler, E. Turner, A. Villaluenga, S. Gaudzinski-Windheuser, Eds. (Propylaeum, 2020), pp. 67–104.

22. J. C. Svenning, A review of natural vegetation openness in north-western Europe. *Biol. Conserv.* **104**, 133–148 (2002).

23. J. Döring, J. Müller, M. Jörn, in *Strategien zur Regeneration belasteter Agrarökosysteme des mitteldeutschen Schwarzerdegebietes,* M. Körschens, E.-G. Mahn, Eds. (BG Teubner Verlagsgesellschaft Stuttgart/Leipzig, 1995), pp. 533–568.

24. D. Mania, in *Neumark-Gröbern: Beiträge zur Jagd des Mittelpaläolithischen Menschen,* D. Mania, M. Thomae, T. Litt, T. Weber, Eds. (Veröffentlichungen des Landesamtes für Denkmalpflege und Archäologie Sachsen-Anhalt 43. Landesmuseum für Vorgeschichte, 1990), pp. 9–130.

25. S. Gaudzinski-Windheuser, W. Roebroeks, *Multidisciplinary Studies of the Middle Palaeolithic record from Neumark-Nord* (*Germany*). (Veröffentlichungen des Landesamtes für Denkmalpflege und Archäologie Sachsen-Anhalt 69. Landesmuseum für Vorgeschichte, 2014).

26. E. Pop, C. Bakels, Semi-open environmental conditions during phases of hominin occupation at the Eemian Interglacial basin site Neumark-Nord 2 and its wider environment. *Quat. Sci. Rev.* **117**, 72–81 (2015).

27. E. Pop, W. Kuijper, E. van Hees, G. Smith, A. García-Moreno, L. Kindler, S. Gaudzinski-Windheuser, W. Roebroeks, Fires at Neumark-Nord 2, Germany: An analysis of fire proxies from a Last Interglacial Middle Palaeolithic basin site. *J. F. Archaeol.* **41**, 603–617 (2016).

28. M. J. Sier, W. Roebroeks, C. C. Bakels, M. J. Dekkers, E. Brühl, D. De Loecker, S. Gaudzinski-Windheuser, N. Hesse, A. Jagich, L. Kindler, W. J. Kuijper, T. Laurat, H. J. Mücher, K. E. H. Penkman, D. Richter, D. J. J. van Hinsbergen, Direct terrestrial-marine correlation demonstrates surprisingly late onset of the last interglacial in central Europe. *Quatern. Res.* **75**, 213–218 (2011).

29. H. Müller, Pollenanalytische Untersuchungen und Jahresschichtenzählungen an der holsteinzeitlichen Kieselgur von Münster-Breloh. *Geol. Jahrb.* **A21**, 107–140 (1974).

30. B. Menke, R. Tynni, Das Eeminterglazial und das Weichselfrühglazial von Rederstall/Dithmarschen und ihre Bedeutung für die mitteleuropäische Jungpleistozän-Gliederung. *Geol. Jahrbuch. R. A, Allg. und Reg. Geol. BR Deutschl. und Nachbargebiete, Tektonik, Stratigr. Paläontologie,* 3–120 (1984).

31. D. Mania, M. Altermann, G. Böhme, T. Böttger, E. Brühl, H.-J. Dohle, K. Erd, K. Fischer, R. Fuhrmann, W.-D. Heinrich, R. Grube, P. G. Karelin, J. Koller, K. V. Kremenetski, T. Laurat, J. Van der Made, D. H. Mai, U. Mania, R. Musil, T. Pfeiffer-Deml, E. Pietrzeniuk, T. Schüler, M. Seifert-Eulen, M. Thomae, *Neumark-Nord. Ein interglaziales Ökosystem des mittelpaläolithischen Menschen* (Landesamt für Denkmalpflege und Archäologie Sachsen-Anhalt, 2010).

32. D. Mania, M. Thomae, *Zur Geologie und Stratigraphie der pleistozänen Becken von Neumark-Nord (Geiseltal)* (Veröffentlichungen des Landesamtes für Denkmalpflege und Archäologie Sachsen-Anhalt, 2013).

33. S. Gaudzinski-Windheuser, E. S. Noack, E. Pop, C. Herbst, J. Pfleging, J. Buchli, A. Jacob, F. Enzmann, L. Kindler, R. Iovita, M. Street, W. Roebroeks, Evidence for close-range hunting by last interglacial Neanderthals. *Nat. Ecol. Evol.* **2**, 1087–1092 (2018).

34. M. Meyer, E. Palkopoulou, S. Baleka, M. Stiller, K. E. H. Penkman, K. W. Alt, Y. Ishida, D. Mania, S. Mallick, T. Meijer, H. Meller, S. Nagel, B. Nickel, S. Ostritz, N. Rohland, K. Schauer, T. Schüler, A. L. Roca, D. Reich, B. Shapiro, M. Hofreiter, Palaeogenomes of Eurasian straight-tusked elephants challenge the current view of elephant evolution. *eLife* **6**, e25413 (2017).

35. S. Wansa, J. Strahl, I. Rappsilber, in *Multidisciplinary Studies of the Middle Palaeolithic Record from Neumark-Nord (Germany)*, S. Gaudzinski-Windheuser, W. Roebroeks, Eds. (Veröffentlichungen des Landesamtes für Denkmalpflege und Archäologie Sachsen-Anhalt 69. Landesmuseum für Vorgeschichte, 2014), pp. 47–68.

36. H. J. Mücher, in *Multidisciplinary Studies of the Middle Palaeolithic Record from Neumark-Nord (Germany)*, S. Gaudzinski-Windheuser, W. Roebroeks, Eds. (Veröffentlichungen des Landesamtes für Denkmalpflege und Archäologie Sachsen-Anhalt 69. Landesmuseum für Vorgeschichte, 2014), pp. 39–46.

37. C. Bakels, Non-pollen palynomorphs from the Eemian pool Neumark-Nord 2: Determining water quality and the source of high pollen-percentages of herbaceous taxa. *Rev. Palaeobot. Palynol.* **186**, 58–61 (2012).

38. C. Bakels, in *Multidisciplinary studies of the Middle Palaeolithic record from Neumark-Nord (Germany)*, S. Gaudzinski-Windheuser, W. Roebroeks, Eds. (Veröffentlichungen des Landesamtes für Denkmalpflege und Archäologie Sachsen-Anhalt 69. Landesmuseum für Vorgeschichte, 2014), pp. 97–107.

39. W.-D. Heinrich, in *Multidisciplinary studies of the Middle Palaeolithic record from Neumark-Nord (Germany)*, S. Gaudzinski-Windheuser, W. Roebroeks, Eds. (Veröffentlichungen des Landesamtes für Denkmalpflege und Archäologie Sachsen-Anhalt 69. Landesmuseum für Vorgeschichte, 2014), pp. 137–142.

40. W. J. Kuijper, in *Multidisciplinary Studies of the Middle Palaeolithic Record from Neumark-Nord (Germany)*, S. Gaudzinski-Windheuser, W. Roebroeks, Eds. (Veröffentlichungen des Landesamtes für Denkmalpflege und Archäologie Sachsen-Anhalt 69. Landesmuseum für Vorgeschichte, 2014), pp. 79–97.

41. S. Milano, E. Pop, W. Kuijper, W. Roebroeks, S. Gaudzinski-Windheuser, K. Penkman, L. Kindler, K. Britton, Environmental conditions at the Last Interglacial (Eemian) site Neumark-Nord 2, Germany inferred from stable isotope analysis of freshwater mollusc opercula. *Boreas* **49**, 477–487 (2020).

42. E. Pop, C. Bakels, W. Kuijper, H. Mücher, M. van Dijk, The dynamics of small postglacial lake basins and the nature of their archaeological record: A case study of the Middle Palaeolithic site Neumark-Nord 2, Germany. *Geoarchaeology* **30**, 393–413 (2015).

43. M. Seifert-Eulen, in *Veröffentlichungen des Landesamtes für Denkmalpflege und Archäologie Sachsen-Anhalt* (Landesmuseum für Vorgeschichte, 2010), vol. 62, pp. 267–272.

44. J. Strahl, M. R. Krbetschek, J. Luckert, B. Machalett, S. Meng, E. A. Oches, I. Rappsilber, S. Wansa, L. Zöller, Geologie, Paläontologie und Geochronologie des Eem-Beckens Neumark-Nord 2 und Vergleich mit dem Becken Neumark-Nord 1 (Geiseltal, Sachsen-Anhalt). *E&G Quat. Sci. J.* **59**, 120–167 (2010).

45. L. Eissmann, in *Altenburger Naturwissenschaftliche Forschungen*, L. Eissmann, Ed. (Naturkundliches Museum Mauritianum, 1990), vol. 5, pp. 1–301.

46. M. E. Peters, P. E. Higuera, Quantifying the source area of macroscopic charcoal with a particle dispersal model. *Quatern. Res.* **67**, 304–310 (2007).

47. W. Roebroeks, C. C. Bakels, in *Settlement, Society and Cognition in Human Evolution: Landscapes in Mind*, F. Coward, F. Wenban-Smith, M. Pope, R. Hosfield, Eds. (Cambridge University Press, 2015), pp. 174–188.

48. S. Wansa, R. Wimmer, in *Die Eemwarmzeit und die frühe Weichselzeit im Saale-Elbe-Gebiet: Geologie, Paläontologie, Palökologie*, L. Eissmann, Ed. (Altenburger Naturwissenschaftliche Forschungen 5, 1990), pp. 49–91.

49. T. Litt, Pollenanalytische Untersuchungen zur Vegetations-und Klimaentwicklung während des Jungpleistozäns in den Becken von Gröbern und Grabschütz. *Altenburg. Naturwissenschaftliche Forschungen.* **5**, 92–105 (1990).

50. T. Litt, in *Neumark-Gröbern: Beiträge Zur Jagd Des Mittelpaläolithischen Menschen,* D. Mania, M. Thomae, T. Litt, T. Weber, Eds. (Veröffentlichungen des Landesamtes für Denkmalpflege und Archäologie Sachsen-Anhalt 43. Landesmuseum für Vorgeschichte, 1990), pp. 193–208.

51. A. A. Velichko, E. Y. Novenko, V. V. Pisareva, E. M. Zelikson, T. Boettger, F. W. Junge, Vegetation and climate changes during the Eemian interglacial in Central and Eastern Europe: Comparative analysis of pollen data. *Boreas.* **34**, 207–219 (2005).

52. W. H. Zagwijn, An analysis of Eemian climate in Western and Central Europe. *Quat. Sci. Rev.* **15**, 451–469 (1996).

53. B. Bratlund, in *The Role of Early Humans in the Accumulation of European Lower and Middle Palaeolithic Bone Assemblages*, S. Gaudzinski, E. Turner, Eds. (Habelt Verlag Bonn, 1999), pp. 255–262.

54. S. Sugita, M.-J. Gaillard, A. Broström, Landscape openness and pollen records: a simulation approach. *The Holocene* **9**, 409–421 (1999).

55. M. Seifert, in *Beiträge zur Jagd des mittelpaläolithischen Menschen*, D. Mania, M. Thomae, T. Litt, T. Weber, Eds. (Veröffentlichungen des Landesamtes für Denkmalpflege und Archäologie Sachsen-Anhalt 43. Landesmuseum für Vorgeschichte, 1990), pp. 149–158.

56. D. Mania, Zur Paläontologie des Interglazials von Neumark-Nord im Geiseltal. *Praehistoria Thuringica* **4**, 67–94 (2000).

57. D. H. Mai, in *Neumark-Gröbern: Beiträge Zur Jagd Des Mittelpaläolithischen Menschen*, D. Mania, M. Thomae, T. Litt, T. Weber, Eds. (Veröffentlichungen des Landesamtes für Denkmalpflege und Archäologie Sachsen-Anhalt 43. Landesmuseum für Vorgeschichte, 1990), pp. 159–160.

58. K. Britton, S. Gaudzinski-Windheuser, W. Roebroeks, L. Kindler, M. P. Richards, Stable isotope analysis of well-preserved 120,000-year-old herbivore bone collagen from the Middle Palaeolithic site of Neumark-Nord 2, Germany reveals niche separation between bovids and equids, *Palaeogeogr. Palaeoclimatol. Palaeoecol.* **333-334**, 168–177 (2012).

59. K. Britton, S. Pederzani, L. Kindler, W. Roebroeks, S. Gaudzinski-Windheuser, M. P. Richards, T. Tütken, Oxygen isotope analysis of Equus teeth evidences early Eemian and early Weichselian palaeotemperatures at the Middle Palaeolithic site of Neumark-Nord 2, Saxony-Anhalt, Germany. *Quat. Sci. Rev.* **226**, 106029 (2019).

60. J. Döring, H. Borg, Ist das Klima von Halle (Saale) noch „normal”? Betrachtungen anhand der Temperatur- und Niederschlagsreihe von 1851 bis heute. *Hercynia* **41**, 3–21 (2008).

61. T. D. Price, M. J. Spicuzza, I. J. Orland, J. W. Valley, Instrumental investigation of oxygen isotopes in human dental enamel from the Bronze Age battlefield site at Tollense, Germany. *J. Archaeol. Sci.* **105**, 70–80 (2019).

62. N. Kühl, T. Litt, Quantitative time series reconstruction of Eemian temperature at three European sites using pollen data. *Veg. Hist. Archaeobot.* **12**, 205–214 (2003).

63. N. Kühl, T. Litt, C. Schölzel, A. Hense, Eemian and Early Weichselian temperature and precipitation variability in northern Germany. *Quat. Sci. Rev.* **26**, 3311–3317 (2007).

64. N. Benecke, G. Böhme, W. D. Heinrich, Wirbeltierreste aus interglazialen Beckensedimenten von Gröbern (Kr. Gräfenhainichen) und Grabschütz (Kr. Delitzsch). *Altenburg. Naturwiss. Forsch.* **5**, 231–281 (1990).

65. W. Soergel, *Der Rabutzer Beckenton: Geologie*, *Paläontologie*, *Biologie* (Veröffentlichungen Provinzialmus, 1920).

66. W. J. Ripple, R. L. Beschta, Trophic cascades in Yellowstone: The first 15years after wolf reintroduction. *Biol. Conserv.* **145**, 205–213 (2012).

67. B. Van Valkenburgh, M. W. Hayward, W. J. Ripple, C. Meloro, V. L. Roth, The impact of large terrestrial carnivores on Pleistocene ecosystems. *Proc. Natl. Acad. Sci. U.S.A.* **113**, 862–867 (2016).

68. D. Mania, D. H. Mai, in *Elefantenreich: eine Fossilwelt in Europa. Begleitband zur Sonderausstellung im Landesmuseum für Vorgeschichte in Halle*, H. Meller, Ed. (Landesamt für Denkmalpflege und Archäologie Sachsen-Anhalt, 2010), pp. 174–185.

69. H. D. Kahlke, Das Pleistozän von Burgtonna in Thüringen. *Quartärpaläontologie.* **3**, 1–399 (1978).

70. D. Mania, M. Altermann, G. Böhme, K. Erd, K. Fischer, W.-D. Heinrich, C. Kremenetzki, J. van der Made, D.-H. Mai, R. Musil, E. Pietrzeniuk, T. Schüler, E. Vlcek, W. Steiner, *Die Travertine in Thüringen und im Harzvorland* (Hall. Jb. Geowiss. Reihe B, Beiheft 17: 83 S., 2003).

71. C. A. Weber, in *Veröffentlichungen des Provinzialmuseums zu Halle* (1920), p. Band I, Heft IV: 3-7.

72. V. Toepfer, Steingeräte und Palökologie der mittelpaläolithischen Fundstelle Rabutz bei Halle (Saale). *Jahresschr. mitteldeutsche Vor.* **41**, 140–177 (1958).

73. K. Erd, Pollenstratigraphie des interglazialen Beckentons von Rabutz südöstlich Halle/Saale. *Altenburg. Naturwissenschaftliche Forschungen.* **5**, 141–147 (1990).

74. N. Stern, The implications of time-averaging for reconstructing the land-use patterns of early tool-using hominids. *J. Hum. Evol.* **27**, 89–105 (1994).

75. T. Litt, in *Großtiere als Landschaftsgestalter*, O. Schmidt, Ed. (Bayerische Landesanstalt für Wald und Forstwirtschaft, 2000), pp. 50–57.

76. R. C. Power, D. C. Salazar-García, M. Rubini, A. Darlas, K. Harvati, M. Walker, J.-J. Hublin, A. G. Henry, Dental calculus indicates widespread plant use within the stable Neanderthal dietary niche. *J. Hum. Evol.* **119**, 27–41 (2018).

77. J. A. Fellows Yates, I. M. Velsko, F. Aron, C. Posth, C. A. Hofman, R. M. Austin, C. E. Parker, A. E. Mann, K. Nägele, K. W. Arthur, J. W. Arthur, C. C. Bauer, I. Crevecoeur, C. Cupillard, M. C. Curtis, L. Dalén, M. Díaz-Zorita Bonilla, J. C. Díez Fernández-Lomana, D. G. Drucker, E. Escribano Escrivá, M. Francken, V. E. Gibbon, M. R. González Morales, A. Grande Mateu, K. Harvati, A. G. Henry, L. Humphrey, M. Menéndez, D. Mihailović, M. Peresani, S. Rodríguez Moroder, M. Roksandic, H. Rougier, S. Sázelová, J. T. Stock, L. G. Straus, J. Svoboda, B. Teßmann, M. J. Walker, R. C. Power, C. M. Lewis, K. Sankaranarayanan, K. Guschanski, R. W. Wrangham, F. E. Dewhirst, D. C. Salazar-García, J. Krause, A. Herbig, C. Warinner, The evolution and changing ecology of the African hominid oral microbiome. *Proc. Natl. Acad. Sci. U. S. A.* **118**, e2021655118 (2021).

78. A. Shaw, M. Bates, C. Conneller, C. Gamble, M.-A. Julien, J. McNabb, M. Pope, B. Scott, The archaeology of persistent places: The Palaeolithic case of La Cotte de St Brelade, Jersey. *Antiquity* **90**, 1437–1453 (2016).

79. G. Snitker, Identifying natural and anthropogenic drivers of prehistoric fire regimes through simulated charcoal records. *J. Archaeol. Sci.* **95**, 1–15 (2018).

80. N. Dussex, N. Bergfeldt, V. de Anca Prado, M. Dehasque, D. Díez-del-Molino, E. Ersmark, F. Kanellidou, P. Larsson, Š. Lemež, E. Lord, E. Mármol-Sánchez, I. N. Meleg, J. Måsviken, T. Naidoo, J. Studerus, M. Vicente, J. von Seth, A. Götherström, L. Dalén, P. D. Heintzman, Integrating multi-taxon palaeogenomes and sedimentary ancient DNA to study past ecosystem dynamics. *Proc. R. Soc. B Biol. Sci.* **288**, 20211252 (2021).

81. FAO, *Guidelines for soil profile description* (Food and Agriculture Organization of the United Nations, ed. 2, 1977).

82. H. Meller, *Elefantenreich: Eine Fossilwelt in Europa. Begleitband zur Sonderausstellung im Landesmuseum für Vorgeschichte in Halle.* (Landesamt für Denkmalpflege und Archäologie Sachsen-Anhalt, 2010).
